# Modeling and optimization of a modified iron-yoked electromagnetic propulsion system using the gravitational search algorithm

**DOI:** 10.1038/s41598-024-75188-5

**Published:** 2024-10-23

**Authors:** M. Mohamed Magdy, Haitham El-Hussieny, Ahmed M. R. Fath El-Bab, Mahmoud M. M. Abdo, Sabah M. Ahmed

**Affiliations:** 1 Physics and Mathematics Engineering Department, Faculty of Engineering, Ain Shams University, Cairo, 11535 Egypt; 2 Department of Mechatronics and Robotics Engineering, School of Innovative Design Engineering, Egypt-Japan University of Science and Technology, Alexandria, 21934 Egypt; 3 Digital Media Technology Department, Future University in Egypt, Cairo, 11835 Egypt; 4 Electrical Engineering Department, Assiut University, Assiut, 71515 Egypt

**Keywords:** Electromagnetic launcher, Coil gun, Variable inductance, Projectile velocity, Ferromagnetic projectile, Magnetic circuit, Iron yoke, Gravitational search algorithm, Energy science and technology, Engineering, Physics

## Abstract

Potential uses for electromagnetic launchers in defense systems, space exploration, and transportation have recently emerged. In addition, this accelerator has many applications, such as deploying small satellites into low-earth orbit and accelerating high-speed trains (e.g., bullet trains and Hyperloop) with a low-cost propulsion system instead of expensive linear motors, particularly in space applications. Therefore, the full capability and optimization of these launchers’ efficiency are still required. Therefore, this paper focuses on presenting a new design to decrease the coil’s magnetic circuit reluctance and boost the magnetic flux lines by adding a laminated iron yoke surrounding the coil. This design makes the inductance value of the iron-yoked accelerator twice the inductance in case of the absence of the iron-yoke at its peak. Additionally, the initial inductance of the iron-yoked accelerator is approximately 65% higher than that of the coil without the iron yoke. Consequently, the modified design proposed an efficiency of 17.5%, which represents a 60% improvement over the efficiency of the regular accelerator. In addition, the introduced design eliminates the suck-back force using a fast-switching device (IGBT) to switch the coil off when the projectile reaches half of the coil. Moreover, a mathematical model for the iron-yoked accelerator is built on MATLAB Simulink and validated experimentally. An artificial intelligence optimization technique, the gravitational search algorithm (GSA), is used to optimize the accelerator parameters, such as the number of turns, capacitor value, and capacitor voltage. Finally, the experimental evaluation of the GSA-optimized system demonstrated an additional 15% enhancement in efficiency, bringing the total efficiency to 20%.

## Introduction

Today, electromagnetics is a crucial science used to create mechanical forces and torques that launch spacecraft and vehicles into space. It transforms electrical energy into kinetic energy to quickly accelerate a ferromagnetic projectile. Electromagnetic science has benefited numerous applications, such as Earth-to-Orbit (ETO) Microsatellite Systems^1–3^; Powerful launchers that can propel small satellites to settle at low Earth orbit^4,5^; Low-speed and high-speed trains, high-speed, long-range fire support naval guns and direct satellite launch to space^6^; Launch of lunar liquid oxygen (LLOX) from the moon to the stationary Lagrangian point L2^7^; Electromagnetic guns^8^; an electromagnetic launcher for the weft insertion system^9^; and Missile launcher to launch toxic waste into space^10^.

There are many promising applications for this kind of launcher, magnetic levitation vehicles, and hyperloop in remote areas with renewable energy^11^. Moreover, launching satellites, especially small and nanosatellites, as in^12^. In addition, the electromagnetic accelerator removes waste from the International Space Station(ISS) instead of using a cargo ship, as proposed in^9^, because the cost of delivering cargo to the ISS will increase sevenfold in 2021.

The coil electromagnetic accelerator consists of eight main parts as used in^11,13–16^. These parts are the Accelerator coil, Capacitor bank, Voltage-Measuring Module, Switching system, Power supply circuit, Velocity-measuring module, Projectile, and Process control system. The simplicity of the accelerator system components enables it to use renewable energy resources like solar energy^16^. Table 1 displays the procedures and findings of recent research to improve the performance of single-stage reluctance coil launchers.

**Table 1:** The procedures and findings of recent studies aim to improve the efficiency of single-stage coil launchers.

Authors	Year	Modification	Improved efficiency
To eliminate/reduce the suck-back force			
Moshe Einat et al.^13^	2023	They used a bipolar capacitor instead of a unipolar one to allow the charging in the opposite direction	0.597%
C. Liang et al.^17^	2021	They used a resistor consumption technique that successfully curbs the production of reverse force	7.43%
H. min Deng et al.^18^	2020	They used the simulation to indicate when the IGBT turns off	5.95%
To reduce the eddy current			
Hui-min Deng et al.^14^	2022	They examined six kinds of soft magnetic materials and four structures to select the proper ones for the armature They found that the silicon steel radially laminated armature is a better choice for the armature design of the reluctance accelerator In addition, they eliminated the suck-back force by using a pre-determined turn-off time for the IGBT switching device	10.73%
To increase the output velocity			
S. Kim and J. Kim^19^	2022	They present a nine-stage accelerator to accelerate a projectile	2.27%
Sandia National Laboratories^20^	2017	They designed a 960m-long accelerator to launch an 1820-kg package containing a 100-kg satellite and a 650-kg boost rocket for orbital insertion	–

**Fig. 1:**
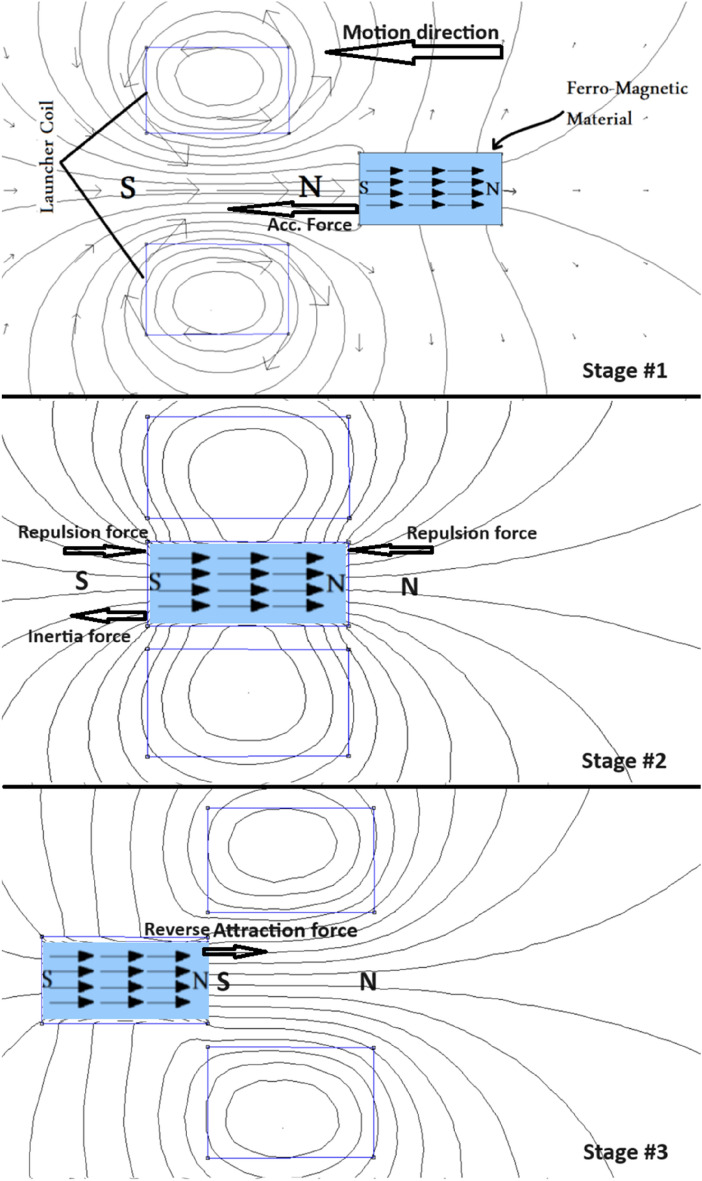
The projectile stages inside the accelerating coil.

The acceleration coil generates magnetic flux lines interacting with the projectile to produce an axial accelerating force. As shown in Fig. 1, the projectile passes through three stages before exiting the barrel. In the first stage, the coil is connected to the capacitor bank to produce magnetic flux lines. Then, an attraction force is developed between the magnetic dipole moments, which are aligned by the magnetic flux lines and the side-facing of the coil (first half of the coil). This force is called the accelerating force. Then, in the second stage, the projectile is wholly connected inside the coil and exposed to three forces, two equal opposing forces between the poles of the coil and the two poles of the projectile. In contrast, the third force is the force of inertia. Finally, the absorption (suck-back) force appears in the third stage. This force is a reverse attractive force towards the center of the coil in the opposite direction to the accelerating force. This force is developed when a magnetic projectile maintaining a magnetic dipole moment (within the projectile material) in one direction is exposed to the opposite face pole of the coil (second half of the coil).

Regarding system modelling, calculating the inductance is challenging because it is difficult to estimate accurately due to many limitations. For example, the fringing effect affects the cross-section area of the air gap around the launcher. Therefore, the authors of^21^ calculated discrete values of the inductance for different positions of the plunger and the coil current using FEMM 4.2 (Finite Element Method Magnetics) simulation to build a linearized inductance look-up table (LUT) block to simulate the system on MATLAB. On the same basis, authors in^22^ used Gauss’s fitting formula to fit the inductance curve obtained by the finite element model built using ANSOFT/Maxwell and get an equation of the launcher inductance as a function of projectile position. All the previously used methods cause the model to lose its flexibility for design and optimization purposes, and the launcher must be designed randomly first.

Optimizing complex systems through traditional trial-and-error methods is often inefficient and time-consuming. To address these challenges, various artificial intelligence-based optimization techniques have been developed, such as genetic algorithm^23^, DSolving^24^, adaptive disturbance observer-based control^25^, and multi-fidelity surrogate-assisted metaheuristics^26^. These methods have demonstrated effectiveness in domains such as wireless power systems^27^, motor control^28^, turbocharger design^29^, UAV system identification^30^, and harmonic suppression in inverters^31^, though each employs different optimization strategies. In^32^, the Cheetah Optimizer (CO) was used to optimize truss structures, effectively balancing objectives like weight reduction and structural integrity.

The gravitational search algorithm (GSA) and the Cheetah Optimiser were compared in this work. Inspired by the cheetah’s hunting strategy, the Cheetah Optimiser is incredibly effective at balancing exploration and exploitation because it employs a phased approach that involves searching, waiting, attacking, and retreating. Though applicable, the Cheetah Optimiser has restrictions in some situations. Large-scale, high-dimensional optimisation tasks are a strong suit for GSA. Because it utilizes mass interactions and gravitational forces to guide solutions. Studies^33^ show that GSA outperformed other techniques like genetic algorithms, consistently showing faster convergence and more accurate results.

This paper employs the gravitational search algorithm (GSA) to optimize the coil launcher system parameters. However, the Cheetah Optimizer offers a robust framework for solving optimization problems, particularly in structurally complex systems. The decision to use GSA is based on the unsatisfactory results obtained with the modified system when using CO.

This paper introduces a new launcher design to boost the number of flux lines. The idea is based on adding a soft iron yoke surrounding the launcher coil. However, the modified design has a higher mass than the accelerator without an iron yoke; the efficiency and the output velocity increase significantly. In addition, the modified design defines the magnetic path of the flux lines, which assists in getting a MATLAB model that is smoother than the ordinary launcher structure. Hence, a modified Gauss’s fitting formula is developed to get the inductance as a function in the launcher’s geometrical dimensions and the projectile’s position. In other words, this formula is not limited to specific data obtained from an experiment or even a finite element model. The obtained MATLAB model is experimentally verified and examined. The modified design characteristics are investigated and shown in this work. Moreover, to reinforce the system’s performance, GSA selects the proper parameters, proving its superiority to the CO.

This paper is structured as follows. Section “Introduction” introduces the system application and reviews relevant previous work. Section “Description of the accelerator system” describes the new launcher design and details the system components. Section “System modeling” presents the mathematical and SIMULINK models for the proposed design, including the electrical circuit and the entire system. Section “Model verification” validates the mathematical and SIMULINK models through 101 experimental tests, covering the induction equation, induction derivative, and velocity tests. Section “Optimization techniques” discusses the optimization algorithms, specifically the Cheetah Optimizer and gravitational search algorithm. Section “Experimental results and discussion” presents experimental outcomes demonstrating various system studies, such as the impact of eliminating the suck-back force using IGBT and the effect of the iron-yoked coil on system performance. It also includes comparing the efficiencies of the Genetic Algorithm and gravitational search algorithm in optimizing system parameters, along with the GSA optimization results and their experimental validation. Section “Contributions and the future work” concludes the contributions and the future work of this work. Finally, “Conclusion” concludes the paper and outlines the outcomes.

## Description of the accelerator system

**Fig. 2:**
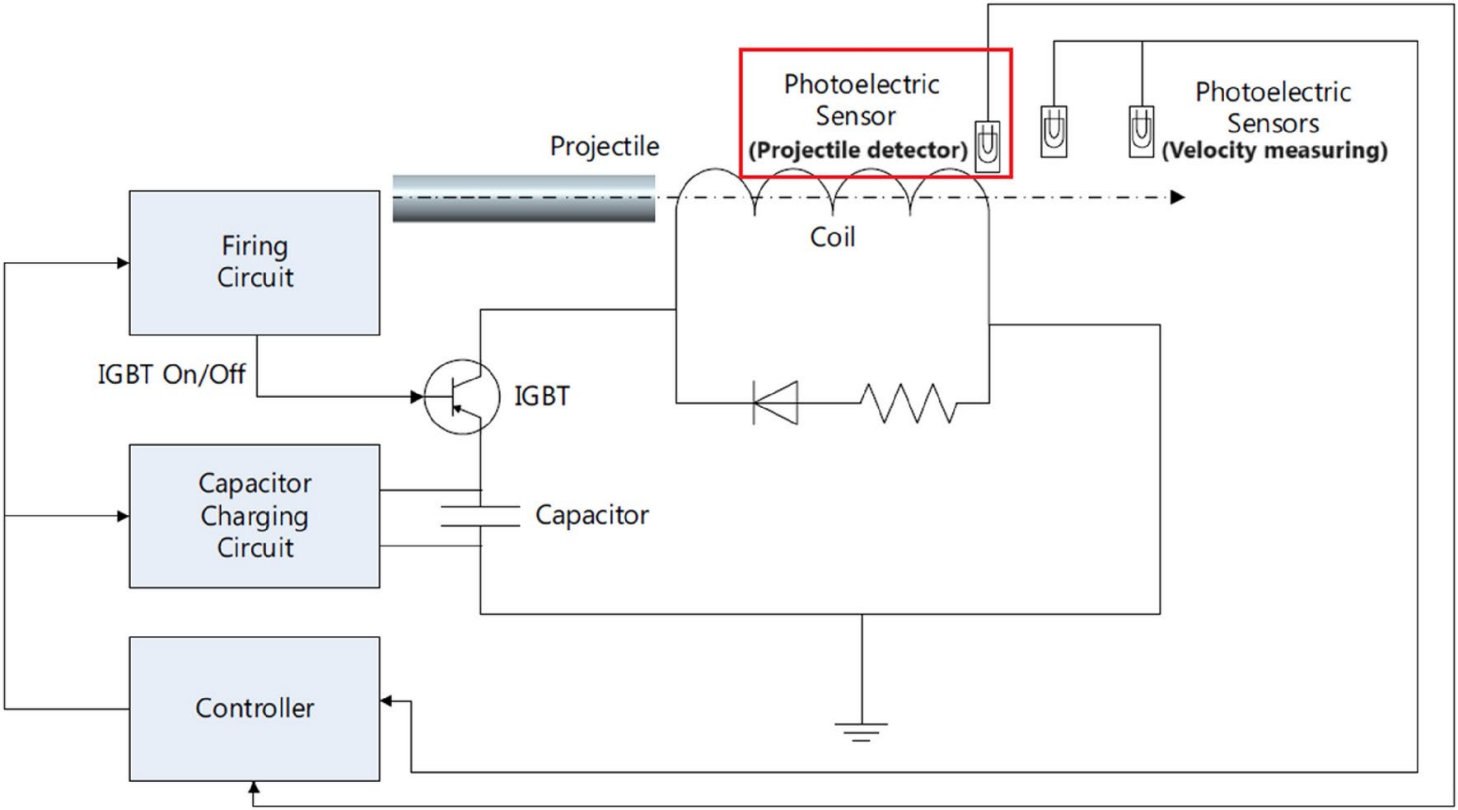
The structure of the entire system.

The system’s components are the accelerator coil, capacitor bank, switching device and its firing circuit, velocity measuring module, projectile, and 133 MHz Dual-core Arm Cortex processor-based control circuit. Figure 2 illustrates the structure of the entire system and the interconnection between its components and modules.

### Accelerator coil

**Fig. 3:**
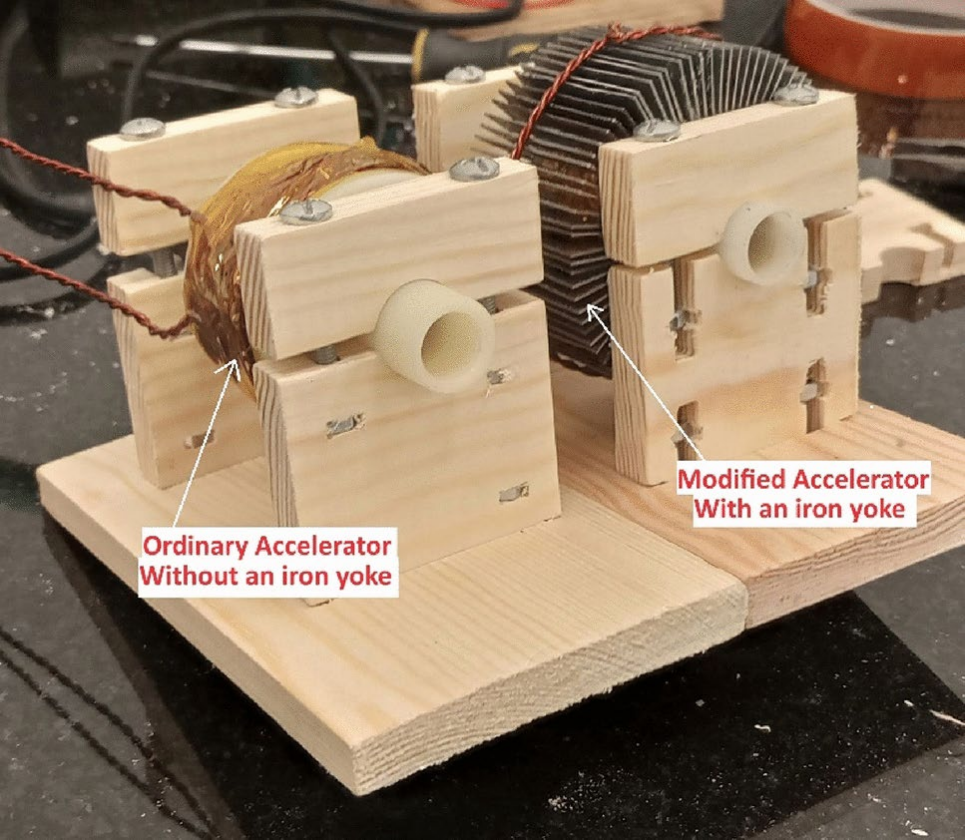
Accelerator coil with/without an iron yoke.

**Fig. 4:**
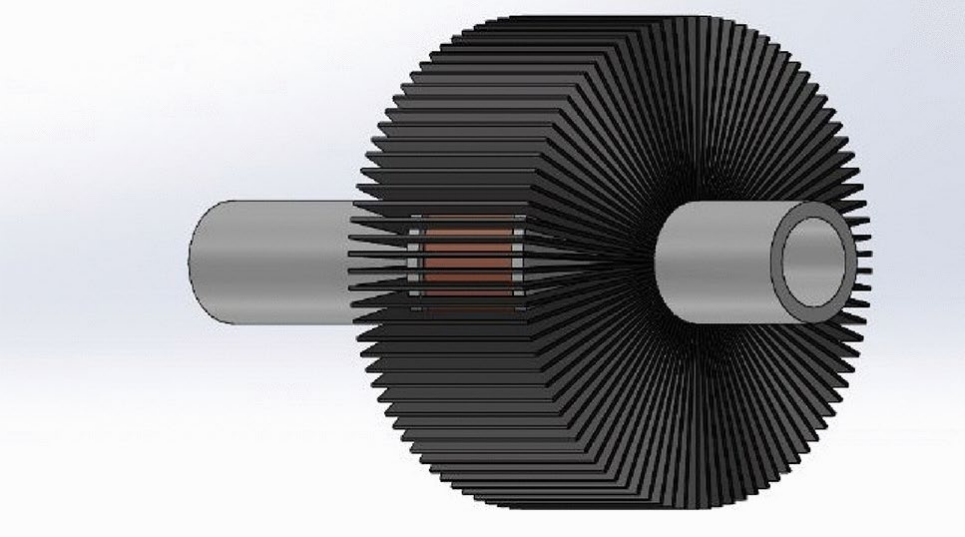
A drawing for the accelerator coil with an iron yoke.

The accelerator coil is the central part of this system. This coil generates lines of magnetic flux, which interact with the magnetic dipoles of the magnetic projectile to accelerate the projectile, as illustrated in^34^. According to Eq. (1), the authors decided to add an iron yoke around the regular accelerator coil to reduce the magnetic reluctance of the air around the coil, as shown in Fig. 3. Equation (1) shows the vital effect of decreasing magnetic impedance in increasing the number of magnetic flux lines. The impact of the iron yoke on the system will be studied experimentally. Figures 3 and 4 show the accelerator structure with and without adding the laminated iron yoke.

1 $$\varphi = \frac{{{\text{NI}}}}{{\Re_{{{\text{total}}}} }}$$

where: $\varphi$: magnetic flux lines within the magnetic material, ${\mathfrak{R}}_{\text{total}}$: Total magnetic reluctance of accelerator magnetic circuit as a function of projectile position. $\text{N}$: Number of turns of accelerator coil, $\text{I}:$ The current passes through the coil.

### Capacitor bank 

**Fig. 5:**
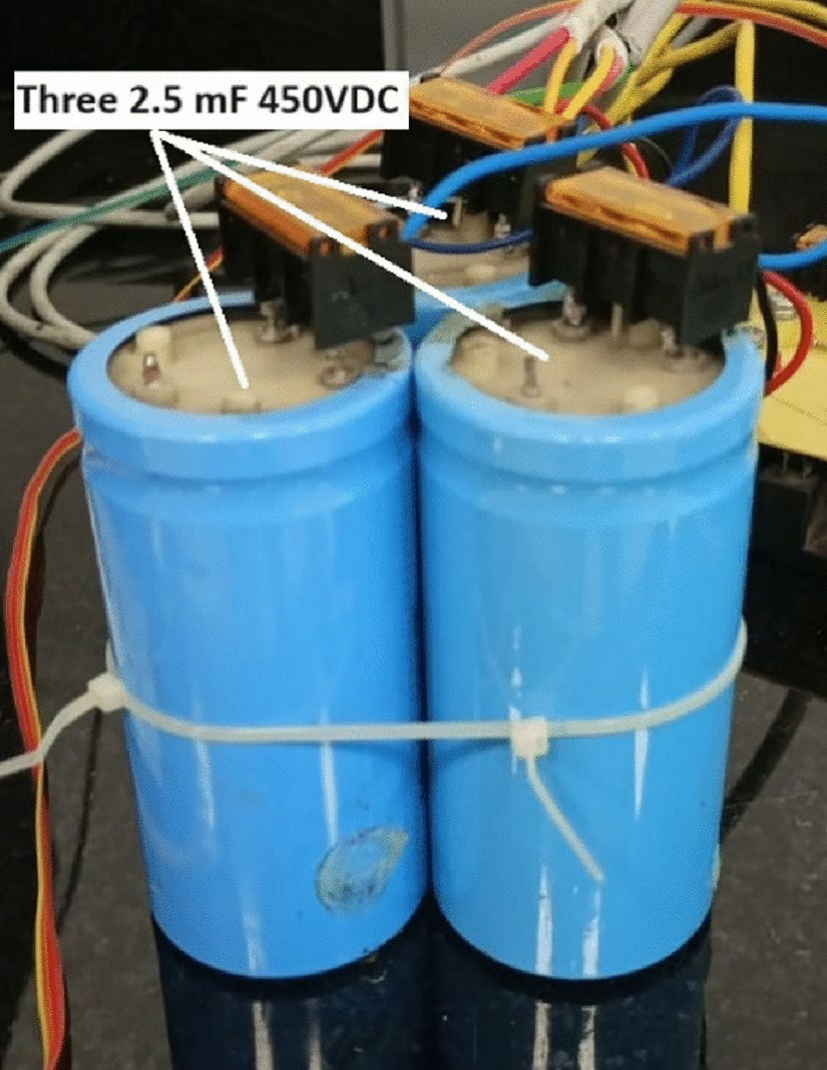
Three identical 2.5 mF capacitors are connected in parallel to get a capacitor bank of 7.5 mF.

A capacitor bank is used to supply an impulsive current waveform when connected to the accelerator coil. The used capacitor bank consists of three identical capacitors to get a capacitor bank of 7.5mF and 450VDC. Each capacitor is a unipolar 2.5mF and 450VDC capacitor as shown in Fig. 5.

### Switching device

**Fig. 6:**
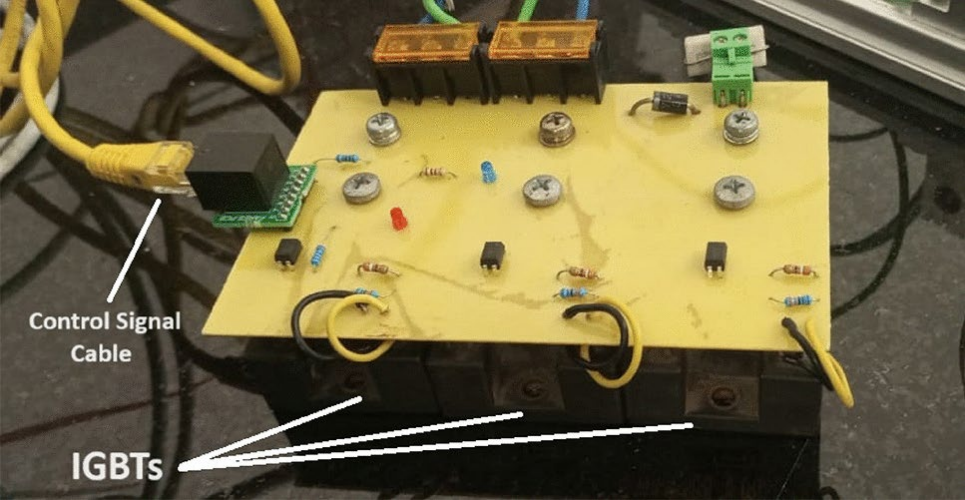
Three cascaded 100A IGBTs connect the capacitor bank with the accelerator coil.

An electronic switching device connects the capacitor bank to the accelerator coil. This device is a High-current highspeed IGBT (2MBI100SC-120_FujiElectric), which provides a high controllability for the control system to switch on/off at an accurate time. The specified IGBT can withstand 100A continuously and 300A for 1 ms. Because the current pulse lasts about 9 ms (depending on the capacitor value and capacitor voltage) with a large current amplitude, the switching device must withstand this current for this period. To achieve that, three consecutive IGBTs are connected in parallel to divide the current. Figure 6 shows the three cascaded 100A IGBTs.

### Velocity measuring module

**Fig. 7:**
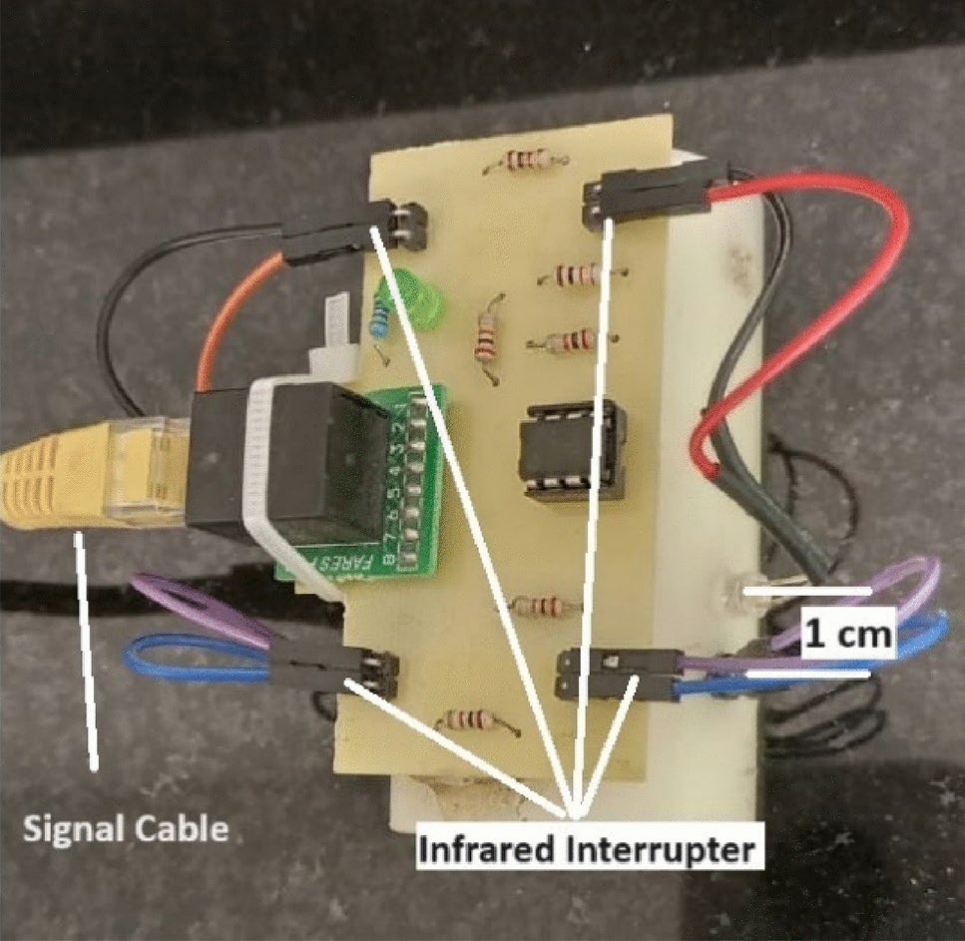
The velocity measurement module is 1 cm apart from two infrared interrupters.

**Fig. 8:**
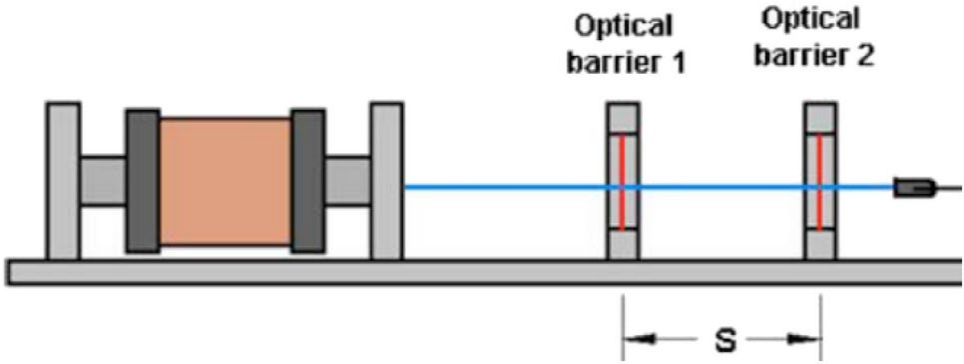
The velocity measurement module.

This module measures the projectile velocity when exiting the accelerator coil. As shown in Eq. (2), its measuring principle depends on measuring the average velocity by dividing an incremental distance by the elapsed time. The incremental distance chosen for this module is 1 cm, as shown in Fig. 7. Two infrared interrupters are used to determine the beginning and the end of this distance. One centimeter is the shortest distance that can be achieved to avoid interference between the two infrared interrupters. Figure 8 shows the conceptual design of this module.

2 $$v = \frac{\Delta x}{{\Delta t}}$$

### Projectile

**Fig. 9:**
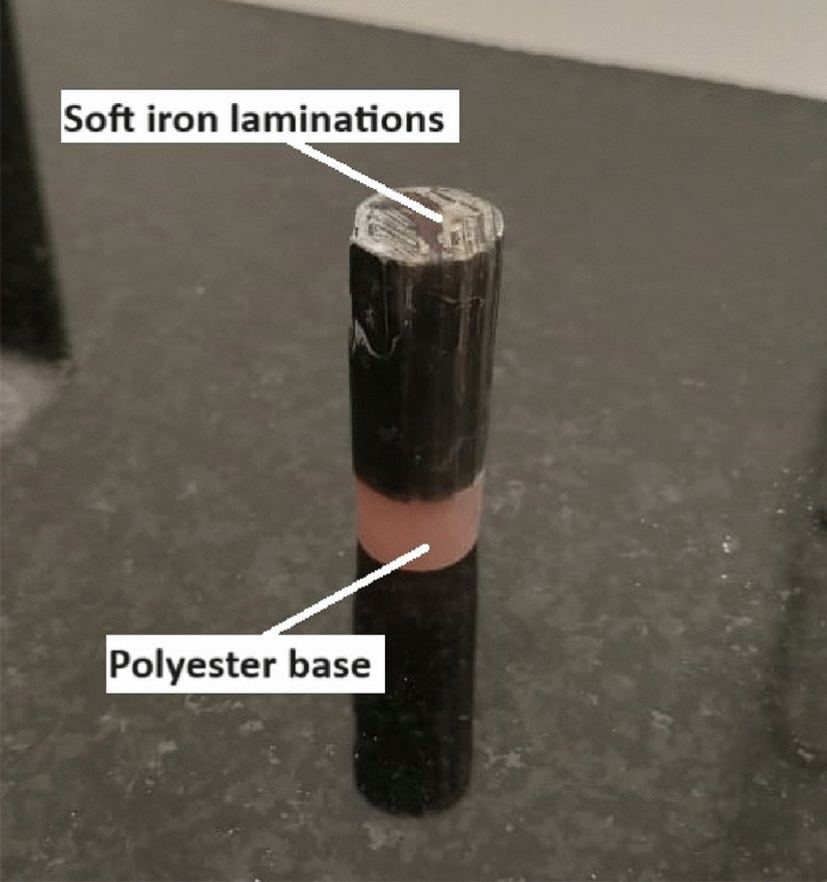
Laminated soft iron projectile encased in a polyester shell.

**Fig. 10:**
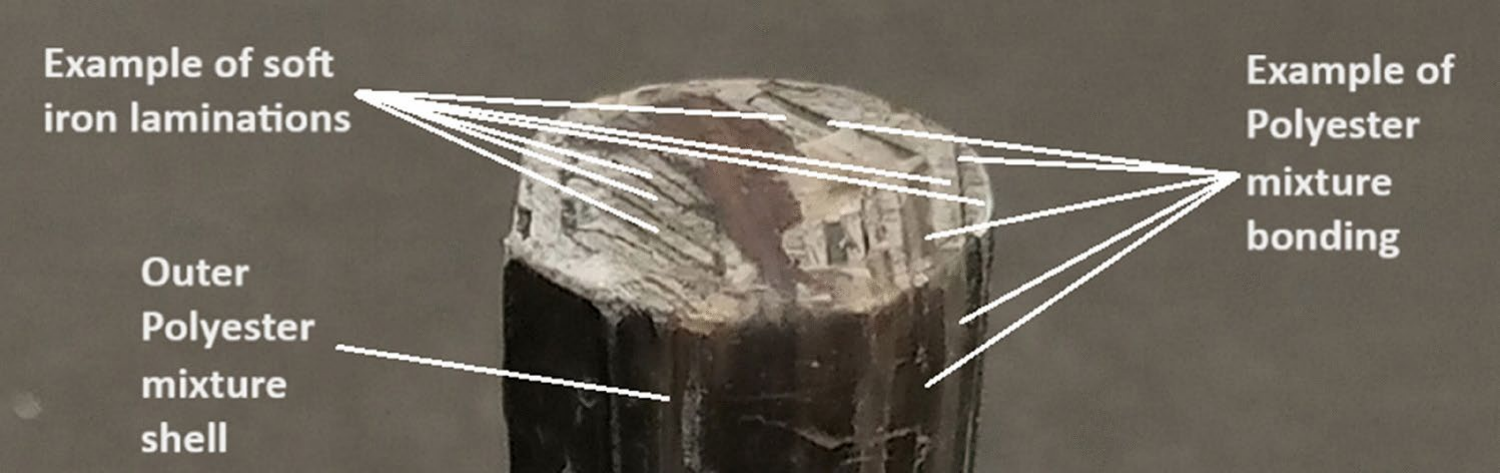
A close view of the projectile structure.

The projectile is the part that interacts with the magnetic flux lines to generate the axial accelerating force. However, many negative forces, such as eddy current force, are produced during the accelerating process. Eddy current reduction depends on increasing the projectile resistance that is achieved by manufacturing the projectile of a high-resistivity material made of laminations, as displayed in Fig. 9. The dimensions of each lamination are as follows: 0.5 mm for thickness, 5 mm for width, and 28 mm for length. In a cylindrical mold, the laminations are stacked vertically to facilitate smooth magnetic flux passage. The laminations are bonded in place by a liquid polyester mixture that is poured into the mold and allowed to solidify. To ensure appropriate curing and bonding, the polyester mixture is formed of 100% polyester plus 2% accelerator and 2% catalyst. Figure 10 provides a close-up view of the projectile’s structure, highlighting the polyester bonds and the vertical arrangement of the laminations.

### Control system 

**Fig. 11:**
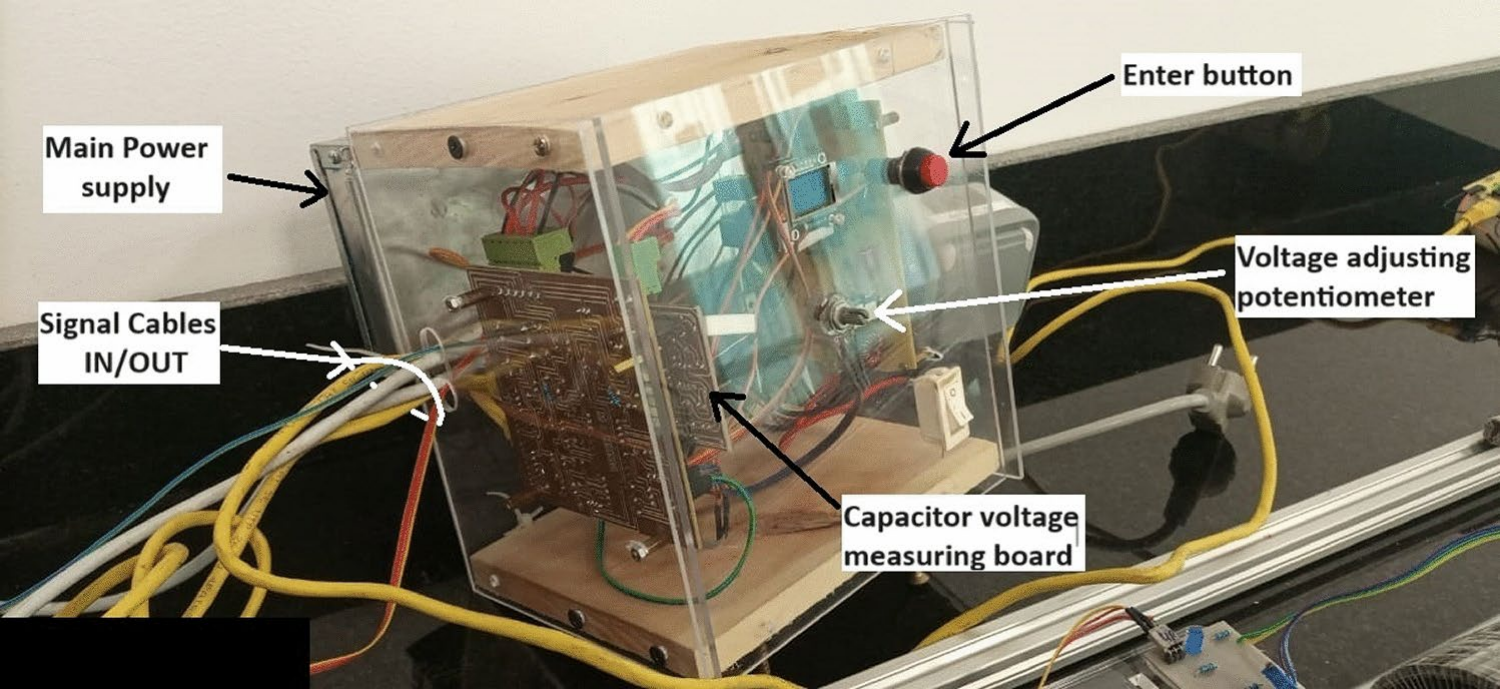
The entire control system.

This unit is the central unit of the accelerator system, controlling every process. The microcontroller used in this control system is powered with a 133 MHz Dual-core Arm Cortex-M0 + processor, 264kB of SRAM, and 2MB of onboard flash memory. Figure 11 shows the entire control system. The processes that can be handled and controlled through this system can be summarized as follows:

1. Get the desired capacitor voltage from the user.
2. Manages the capacitor charging process.
3. When the launch button is pressed, the controller first checks the projectile’s presence to start the launching operation using an infrared interrupter settled on the barrel.
4. The controller switches on the IGBTs (switching device) until the projectile’s centreline coincides with the accelerator coil centreline and switches them off.
5. The velocity measurement module sends signals to the control system to measure the time the projectile travels from the first infrared interrupter to the second one.

**Fig. 12:**
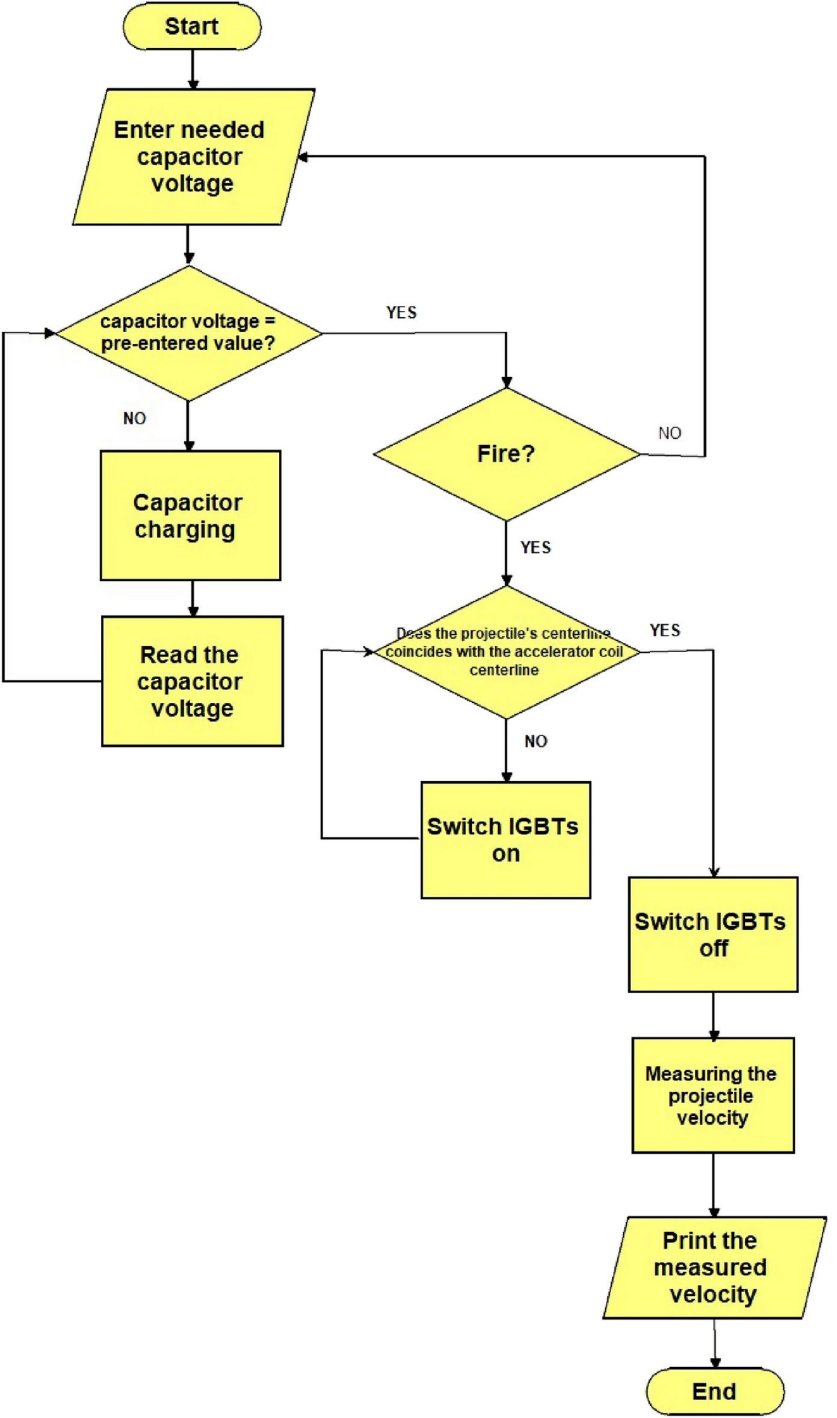
The control system flow chart.

The control system follows the flow chart shown in Fig. 12.

## System modeling

An electromagnetic accelerator is a complex system. Therefore, many parameters should be considered when modeling the entire system. A SIMULINK model is built to simulate the propulsion system.

### Accelerator coil model

The accelerator coil can be modeled in this system as a variable inductance because its inductance value varies with the projectile position inside the coil. The upgraded accelerator coil, shown in Fig. 4, helps model this part more accurately than the ordinary accelerator coil.

It is not accurate to evaluate the inductance of the accelerator as a function in the projectile position L(x) by using Eqs. (3) and (4). This is because of inaccurate determination of the air gap area between the projectile and the iron yoke (around the accelerator) during the projectile motion. Researchers use infinite element software like COMSOL and ANSYS to simulate their models. However, the experimental readings for L(x) show that L(x) follows the Gaussian distribution. Therefore, a modified Gauss fitting formula Eq. (5) is developed to fit the experimental readings by calculating only the maximum and minimum inductance values. The two extremes (L0, Lmax) depend mainly on the construction and shape of the solenoid and can be smoothly calculated according to the system parameters listed in Table 2.

3 $$\Re = \frac{l}{\mu A}$$

where: $\mathfrak{R}$: magnetic reluctance, $l$: length of the path, $A$: area of the core, $\mu$: the permeability of the core

4 $$L\left( x \right) = \frac{{N^{2} }}{{\Re_{{{\text{total}}}} }}$$

where: $\Re_{{{\text{total}}}}$: The total magnetic reluctance of the accelerator magnetic circuit as a function of projectile position.

5 $$L\left( x \right) = \left( {L_{{{\text{max}}}} - L_{0} } \right)e^{{\frac{{ - \left( {x - x_{c} } \right)^{2} }}{{\left( {\frac{{x_{c} }}{2}} \right)^{2} }}}} + L_{0}$$

where: ${L}_{max}$: the maximum value of the accelerator coil inductance ($\mu H$), ${L}_{0}$: the minimum value of the accelerator coil inductance ($\mu H$), ${x}_{c}$: the position at which the projectile engaged totally inside the coil (m).

**Table 2:** The iron-yoked accelerator parameters.

Parameter	Value
Number of turns	165 turns
Coil length ($$w)$$	28 mm
Coil bobbin border thickness	2.5mm
Projectile length	28mm
Projectile cross-section area	117.5 mm^2^
Projectile mass	28.32 g
Laminations number	92
${l}_{short gap}$	5 mm
${l}_{long gap}$	33 mm

Some assumptions about using this method to obtain a mathematical model for the accelerator system exist. The first assumption is that the coil length must equal the projectile length to achieve higher efficiency and smooth calculation, as the initial experimental studies proved. The second assumption is that the air cross-section area inside the coil occupies about ($1-{e}^{-1}$) of the coil’s total area, as demonstrated through many FEMM simulations. The last assumption, the iron yoke, and the projectile reluctances are neglected to simplify the deduced inductance equation. The iron yoke and the projectile reluctances are about many thousand lower than the air gap reluctance due to the higher permeability of the soft iron material than the air gap and the inversely proportional between permeability and the reluctance according to Eq. (3).

**Fig. 13:**
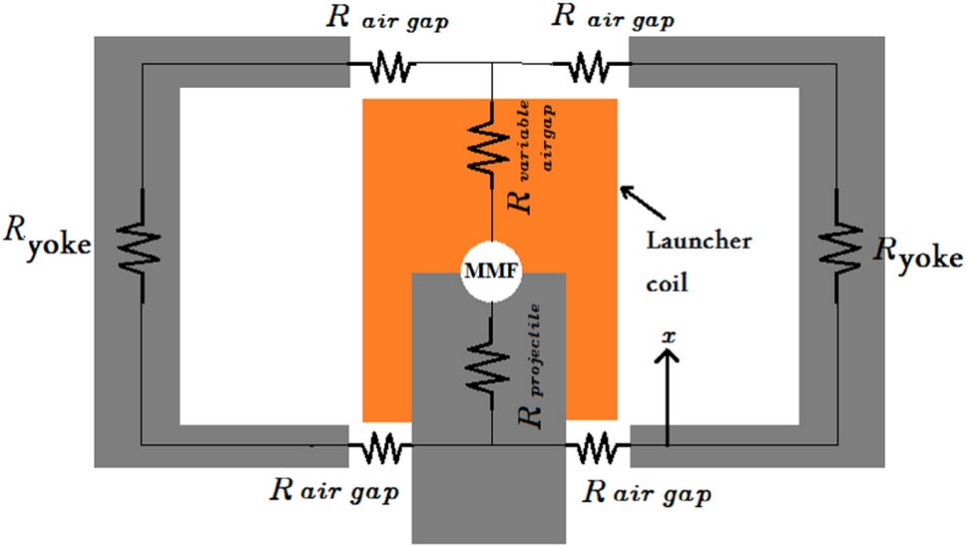
The iron-yoked accelerator magnetic circuit.

**Fig. 14:**
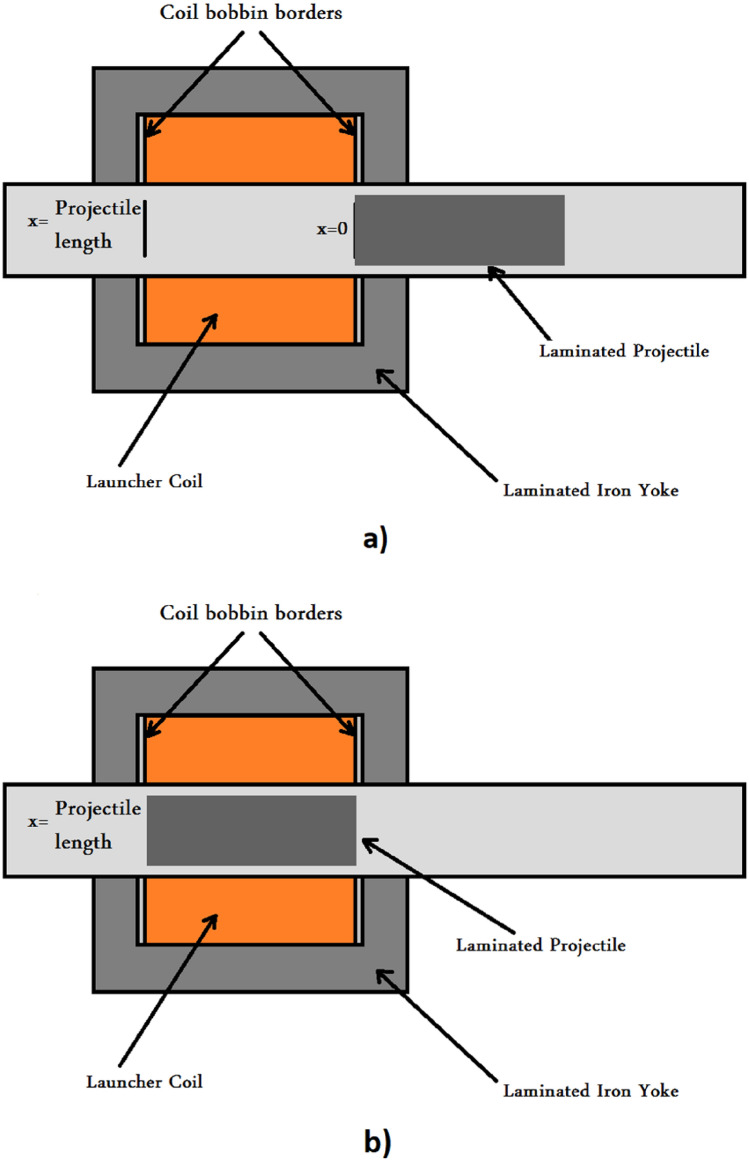
(**a**) A cross-section through the accelerator when the projectile at x = 0. (**b**) A cross-section through the accelerator when the projectile is engaged.

The magnetic circuit illustrated in Fig. 13 shows all magnetic reluctances that are essential to calculate the two extremes (L0, Lmax). L0 is the inductance value when the projectile is at x = 0, as shown in Fig. 14a, and calculated using Eq. (6). Lmax is the coil inductance value when the projectile is engaged totally inside the accelerator coil, as displayed in Fig. 14b and calculated by Eq. (7). Due to the fringing effect, the inductance value will differ from what was expected. Therefore, the fringing factor (Kf) is added to the Lmax inductance equation, which can be calculated using an improved McLyman’s formula^35^ that is shown in Eq. (8). The projectile length is chosen equal to the coil length according to recent research by the same authors^15^. The magnetic saturation is related to the coil current^22^ and its influence on magnetic saturation on the equivalent inductance after exceeding the saturation magnetic flux density. The coil current is not included in the inductance equation to reduce the complexity of the inductance model. Consequently, the model is used until saturation to avoid the mismatch between the simulation and experimental models.

6 $$L_{0} = \frac{{N^{2} \mu_{o} A_{g} }}{{l_{long gap} }}$$

7 $$L_{max} = \frac{{N^{2} \mu_{o} A_{projectile} K_{f} }}{{l_{short gap} }}$$

8 $$K_{f} = 1 + \frac{{q l_{short gap} }}{{\sqrt {A_{projectile} } }} {\text{ln}}\left( {\frac{2w}{{l_{short gap} }}} \right)$$

where: ${\mu }_{o}$: The permeability of the core, ${l}_{short gap}$: Length of the air gap in case the projectile is plugged into the coil (double coil bobbin border thickness), ${l}_{long gap}$: Length of the air gap in case the projectile is out of the coil, ${A}_{g}$: Area of the air gap inside the coil, ${A}_{projectile}$: Axial area of the iron laminations in the projectile, $w$: The Coil length.

### Electrical circuit and accelerating force modelling

**Fig. 15:**
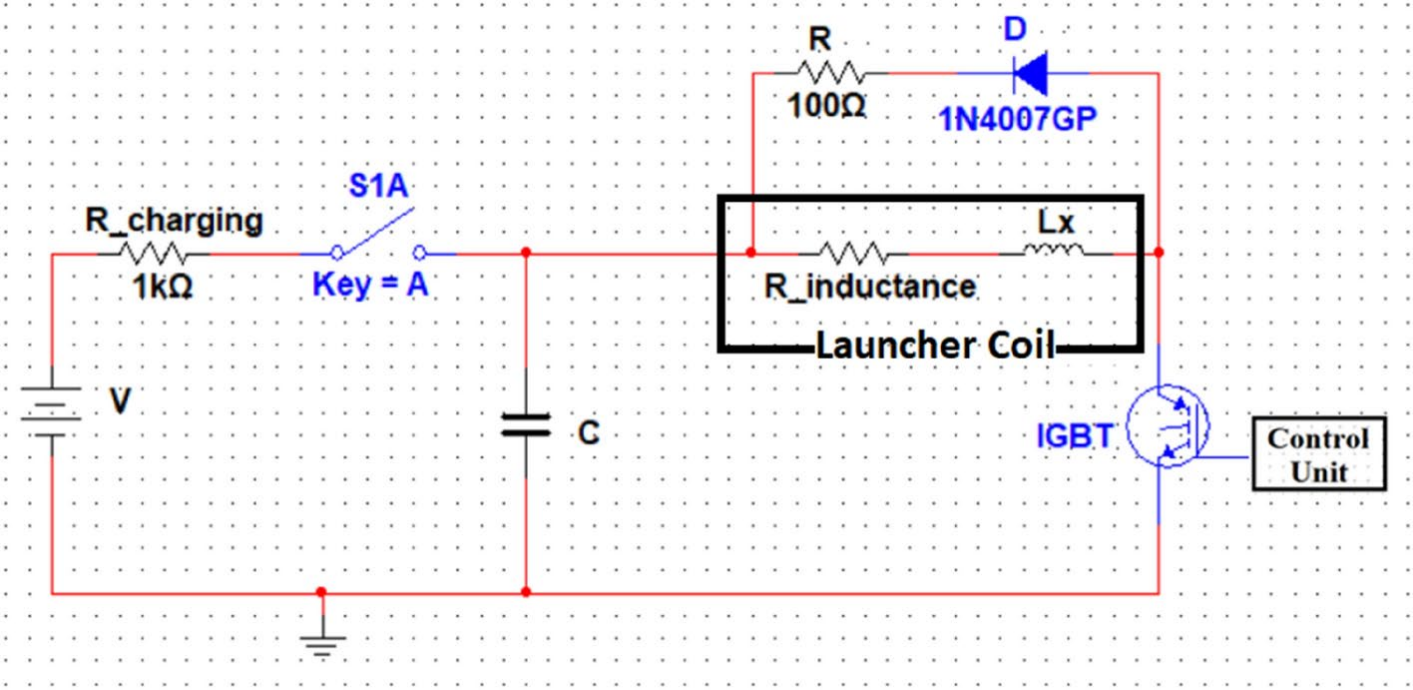
The circuit diagram of the accelerator.

The accelerator electrical system consists of an accelerator coil (an inductor and a resistor), capacitor, and power IGBT, as shown in the schematic Fig. 15. Therefore, it can be simplified to an RLC circuit. The accelerator coil can be modeled as a resistor (R_inductance) in series with a variable inductance concerning the ferromagnetic slug’s position inside the coil, as illustrated in Eq. (5). Applying Kirchhoff’s voltage law (KVL) on the accelerator circuit through the switching-on period can be written as in^22,36^ and shown in Eqs. (9) and (10). The control system turns off the circuit just after the projectile center coincides with the center of the coil. As a result, the RLC circuit becomes an RL circuit. To avoid IGBT damage due to the backward current and to reduce the discharge time of the coil, according to Eq. (13), a series resistor (R) with a diode (D) is connected in parallel with the accelerator, as displayed in Fig. 15.

9 $$V_{C} = V_{R} + \frac{d}{dt}\left( {flux linkages} \right) + V_{IGBT} = V_{R} + \frac{d}{dt}\left( {L\left( x \right) i\left( t \right)} \right) + V_{IGBT}$$

10 $$V_{Co} - \frac{1}{C}\smallint i dt = R i_{L} \left( t \right) + L\left( x \right) \frac{{di_{L} \left( t \right)}}{dt} + i_{L} \left( t \right)\frac{dL\left( x \right)}{{dt}} + V_{IGBT}$$

where $L(x) \frac{d{i}_{L}\left(t\right)}{dt}$ represents the voltage across the inductance, ${i}_{L}\left(t\right)\frac{dL\left(x\right)}{dt}$ is an EMF generated due to the movement of the projectile inside the inductance and can be simplified as

11 $$i_{L} \left( t \right)\frac{dL\left( x \right)}{{dt}} = i_{L} \left( t \right)\frac{dL\left( x \right)}{{dx}} \frac{dx\left( t \right)}{{dt}}$$

where $\frac{dx(t)}{dt}$ is the projectile velocity, $\frac{dL\left(x\right)}{dx}$ represents the derivative of Eq. (5) and should be expressed as

12 $$\frac{dL\left( x \right)}{{dx}} = \left( {L_{max} - L_{0} } \right)\left( {\frac{{ - 2\left( {x - x_{c} } \right)}}{{\left( {\frac{{x_{c} }}{2}} \right)^{2} }}} \right) e^{{\frac{{ - \left( {x - x_{c} } \right)^{2} }}{{\left( {\frac{{x_{c} }}{2}} \right)^{2} }}}}$$

13 $$\tau_{discharge} = \frac{L}{R}$$

Considering the projectile motion, the magnetic energy converted to a mechanical force should be expressed as Eq. (14).

14 $$W = \frac{1}{2}L\left( x \right) i_{L}^{2} \left( t \right)$$

where $W$ is the produced magnetic energy, ${i}_{L}\left(t\right)$ represents the time-dependent excitation current in the coil. The derivative of Eq. (14) on both sides concerning projectile position ($x$) yields an axial electromagnetic force acting on the projectile, as shown in Eq. (15).

15 $$F_{acc.} \left( x \right) = \frac{\partial }{\partial x}\left( {Magnetic Energy} \right) = \frac{\partial }{\partial x}\left( {\frac{1}{2}L\left( x \right) i_{L}^{2} \left( t \right)} \right) = \frac{1}{2} i_{L}^{2} \left( t \right) \frac{dL\left( x \right)}{{dx}}$$

After getting the accelerating force for the projectile, the kinetic equation can be written as

16 $$a\left( x \right) = { }\frac{{F_{acc.} \left( x \right) - f_{drag} \left( v \right)}}{m}$$

where $a(x)$ represents the projectile acceleration hence the projectile velocity can be smoothly evaluated from Eq. (16), $m$ is the projectile mass. ${f}_{drag}(v)$ is the drag force, and it is related to the projectile velocity; it is composed of pressure drag and skin friction drag.

### System delay

All the components used in this system cause a time delay between the switching on and off processes. But three main devices have a huge share in the system delay as follows:

1. The projectile position infrared detector is delayed between detecting the projectile and sending the signal to the microprocessor.
2. The microprocessor causes a delay of about 100 microseconds between receiving the off-trigger signal and producing the off order to the IGBT.
3. The IGBT’s turn-on and turn-off time affect the accelerator’s switching-on period. These values depend on the IGBT’s manufacturing and current rating.

It is mathematically difficult to determine the total delay time. Therefore, this work estimates it experimentally and provides it to the SIMULINK model. The measured delay time is 430 microseconds.

## Model verification

101 tests are conducted to validate the proposed model. Three tests are chosen to cover all aspects of the system because not all system parameters can be measured experimentally.

1. The first test is the inductance equation test, which compares the inductance calculated by the deduced equation with the measured inductance values by a calibrated RLC meter.
2. An inductance derivative test is performed to compare the derivative of the inductance equation to the change of inductance concerning the projectile position experimentally.
3. To examine the entire model, the velocity test is carried out to compare the velocity calculated by the model to the real velocity of the projectile.

### Inductance equation test

**Fig. 16:**
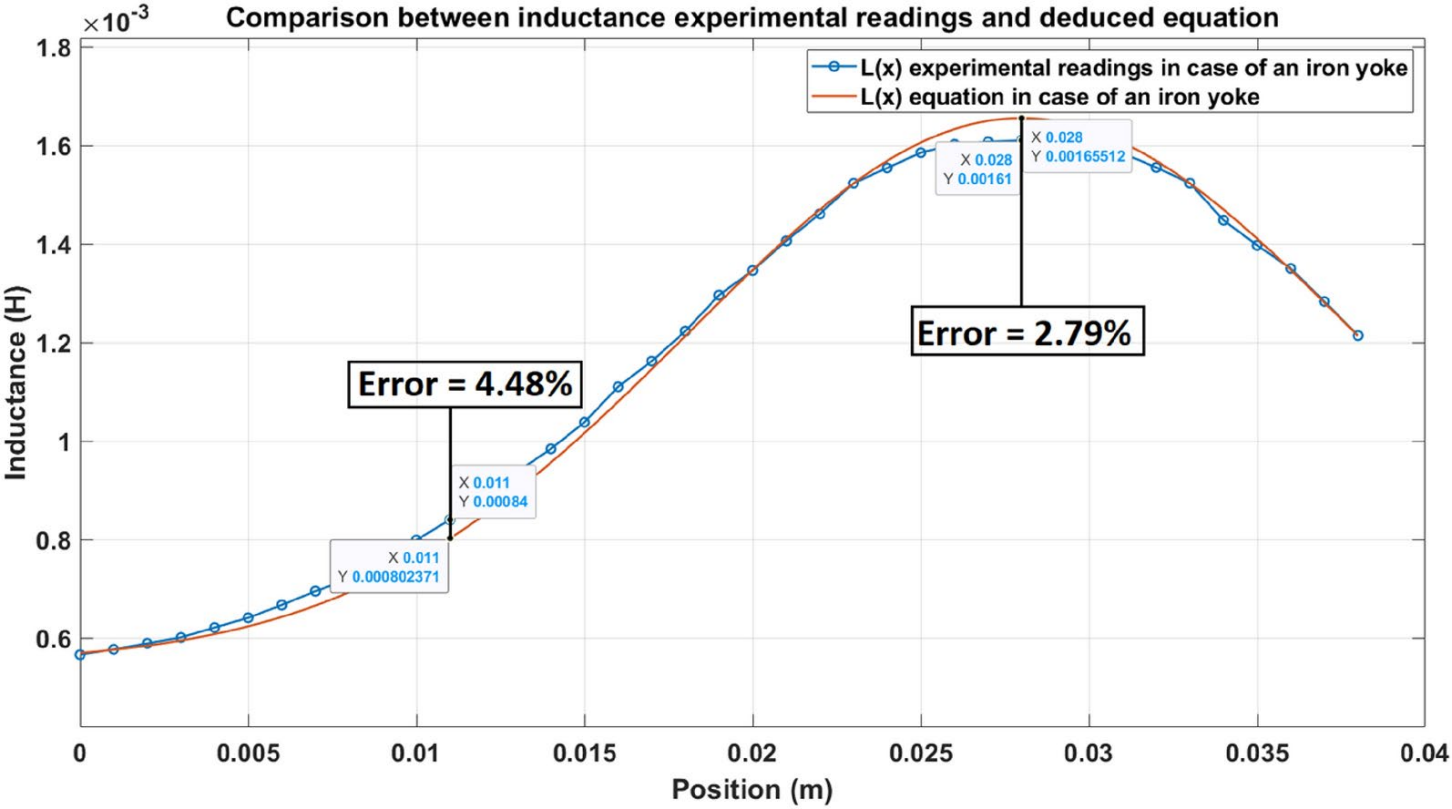
Comparison between inductance experimental readings and deduced equation.

The inductance of the launcher is the most effective part of the launcher model. Therefore, validating that the deduced inductance equation in Eq. (5) gives approximately equal values to the measured inductance readings is crucial. A calibrated RLC meter (Ut58D) measures the inductance experimental readings. Inductance reading is taken for each millimeter displacement of the armature inside the accelerator’s coil, starting from x = 0 till x = 38 mm, as shown in Fig. 14. The results are plotted and shown in Fig. 16.

### Inductance derivative test

**Fig. 17:**
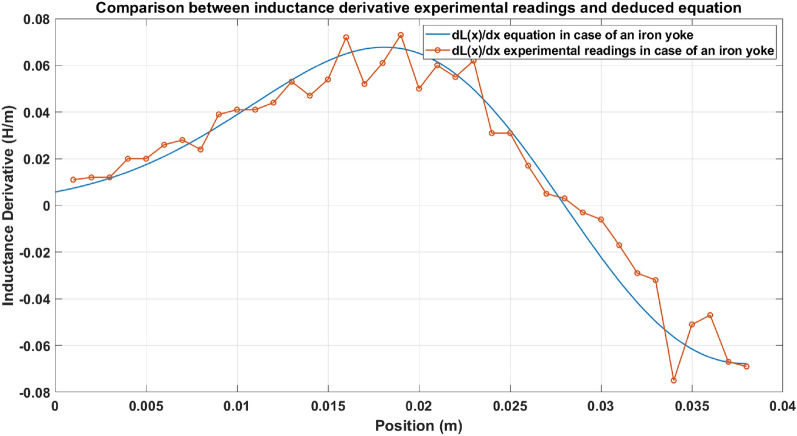
Experimental readings and the deduced equation of inductance’s derivative.

The derivative of the inductance equation ($\frac{dL\left(x\right)}{dx}$) shown in Eq. (12) is an essential parameter in force estimation. A comparison graph is plotted between the experimental readings and the values obtained from the equation and is illustrated in Fig. 17. Figure 17 shows how close a deduced equation is to the true value of the inductance derivative ($\frac{dL\left(x\right)}{dx}$).

### Velocity test

**Fig. 18:**
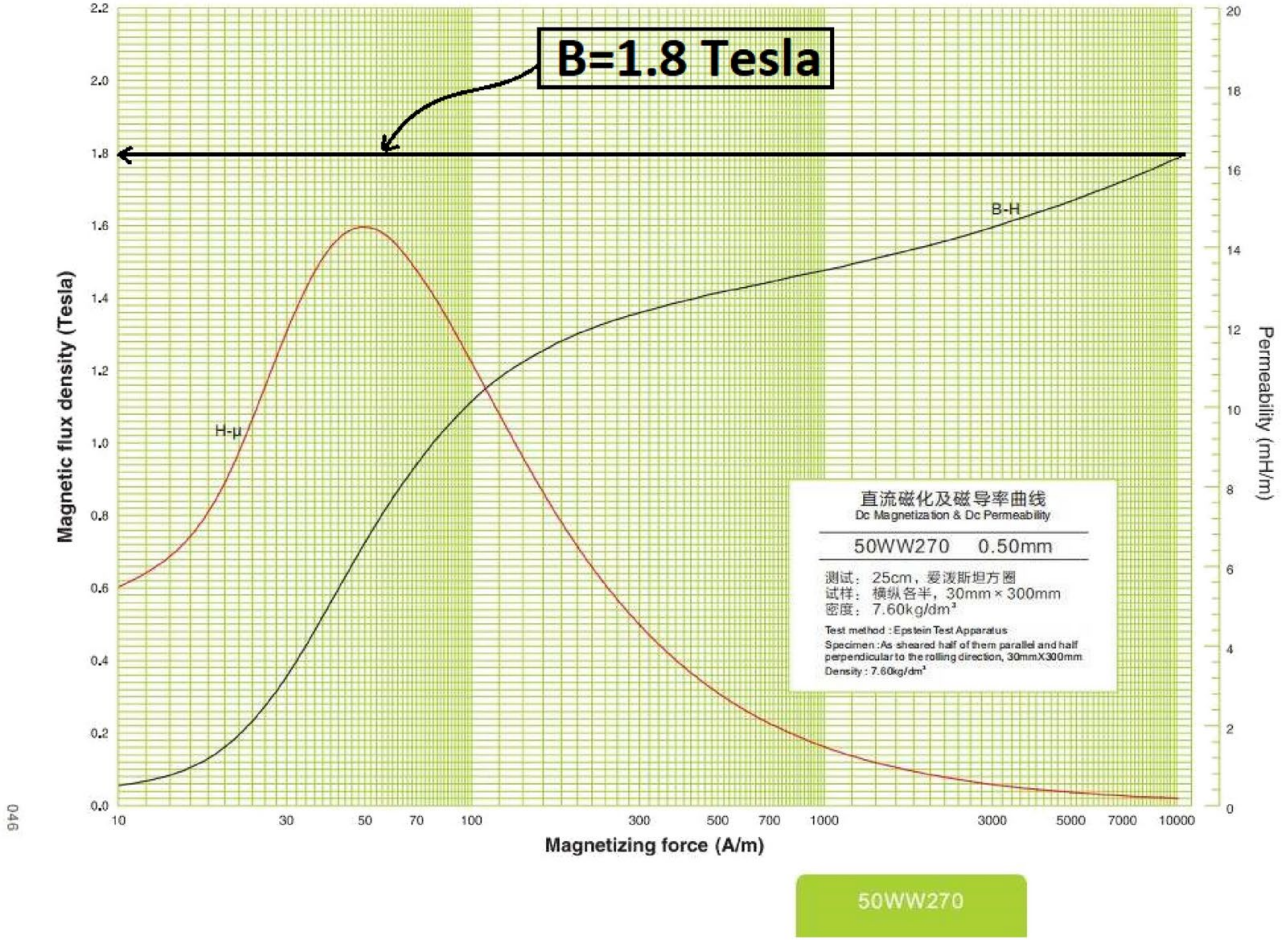
DC magnetization and DC permeability curves of the iron yoke material.

**Fig. 19:**
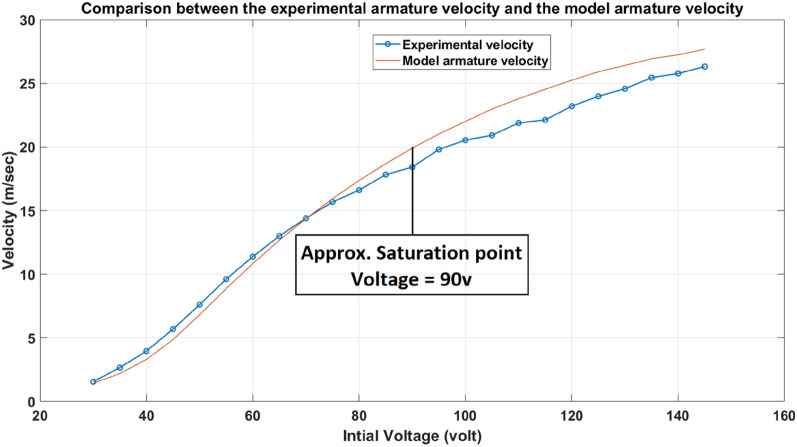
The output velocity of the experimental model and the simulation model of the accelerator.

The propulsion system’s main output is the velocity of the armature at the barrel end. Therefore, the velocity of the armature gained from the SIMULINK model should be compared to the experimental readings of the armature’s velocity at the same voltage. According to Fig. 18, the saturation magnetic field is B = 1.8 T, equivalent to a capacitor voltage of 90 V. Therefore, the two curves in Fig. 19 are approximately identical, with a maximum variance of about 5% until the saturation point, and then the two curves diverge after that point.

## Optimization techniques

Although all the modifications proposed in this work, besides the other research, the optimal parameters and design cannot be gained by the trial-and-error method, particularly for huge systems. Therefore, an artificial intelligence technique is used to get the system parameters that give higher efficiency. The objective function that needs to be maximized is the system efficiency, while the constraints are the magnetic field density and the switching device-rated current.

$$Objective function = MAX\left( {\frac{Projectile kinetic energy}{{Input energy - Remaining energy}}} \right) = MAX\left( { \frac{{\frac{1}{2}mv^{2} }}{{\frac{1}{2}C\left( {V_{intial}^{2} - V_{final}^{2} } \right)}}} \right)$$

The constraints are:

$$\begin{gathered} {\text{Magnetic field denisty}} < 1.8{\text{ T}} \hfill \\ {\text{Coil current}} < 290{\text{A}} \hfill \\ \end{gathered}$$

Two optimization techniques are used. The first one is the Cheetah Optimizer (CO) (commonly used in this field), while the second one is the gravitational search algorithm (GSA).

### Cheetah optimizer

**Fig. 20:**
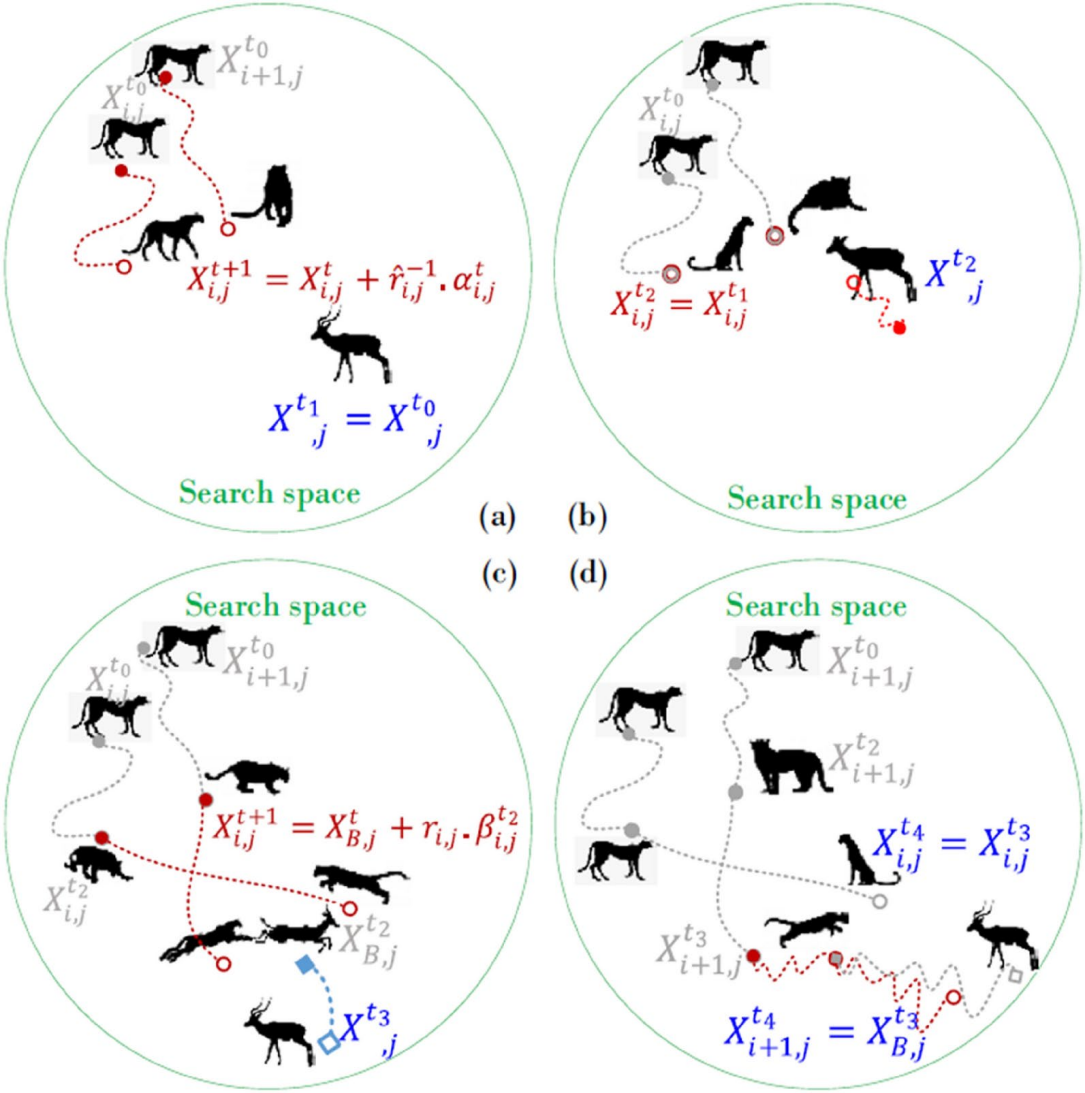
Graphical information of CO’s strategies.

Cheetah Optimizer^37^ is one of the most powerful Artificial intelligence optimization methods. It was presented in a paper written in 2022 by Mohammad Amin Akbari and associates. This algorithm is based on cheetah hunting techniques, which include searching, waiting, attacking, and a further tactic that follows the cheetah’s strategy of going back to a previous location if the prey isn’t found. Large-scale optimization issues have been successfully resolved by the CO algorithm, particularly in the domains of engineering and economic load dispatch optimization. Figure 20 illustrates the search strategy.

### Gravitational search algorithm

**Fig. 21:**
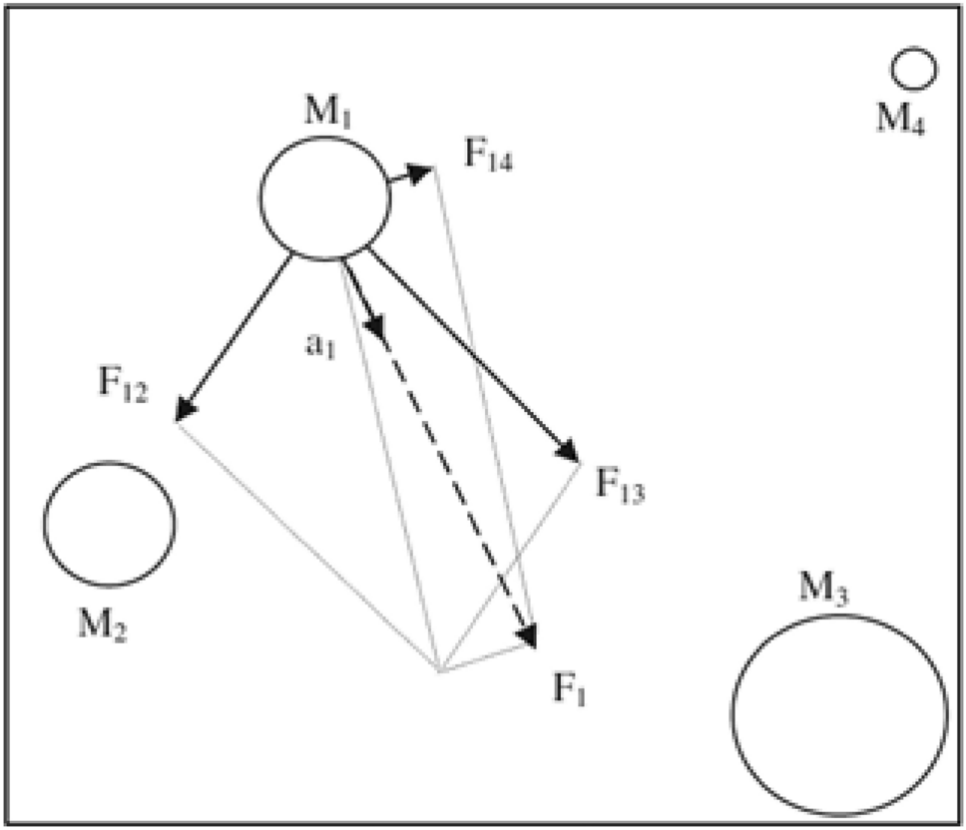
Every mass accelerates in the direction of the resultant force.

The gravitational search algorithm (GSA)^38^ is a population search algorithm proposed by Rashedi et al. in 2009. It is an optimization algorithm based on the law of gravity and mass interactions and Newton’s law of gravitation. All objects attract each other by the gravitational force, and this force causes a movement for all objects in the direction of the objects of heavy masses, as shown in Fig. 21, which illustrates a live example from nature (earth and the moon).

In GSA, each mass is an agent, representing a solution for the problem; each one has four main information: position, inertial mass, active gravitational mass, and passive gravitational mass; all these masses obey:

- Law of Gravity: according to Newton’s law of gravity, each object attracts each other with gravitational force, which between each two objects is directly proportional to the product of their masses and inversely proportional to the distance squared $F=G\frac{{M}_{1} {M}_{2}}{{R}^{2}}$
  where: $G$: Gravitational Constant, ${M}_{1}, {M}_{2}$: First and Second object mass,
  ${R}^{2} :$ Square the distance between the 2 objects.
- Newton’s 2nd Law, which concerns the object’s motion, states that when F is applied to an object, the object starts to move with acceleration $a= \frac{F}{M}$.

**Fig. 22:**
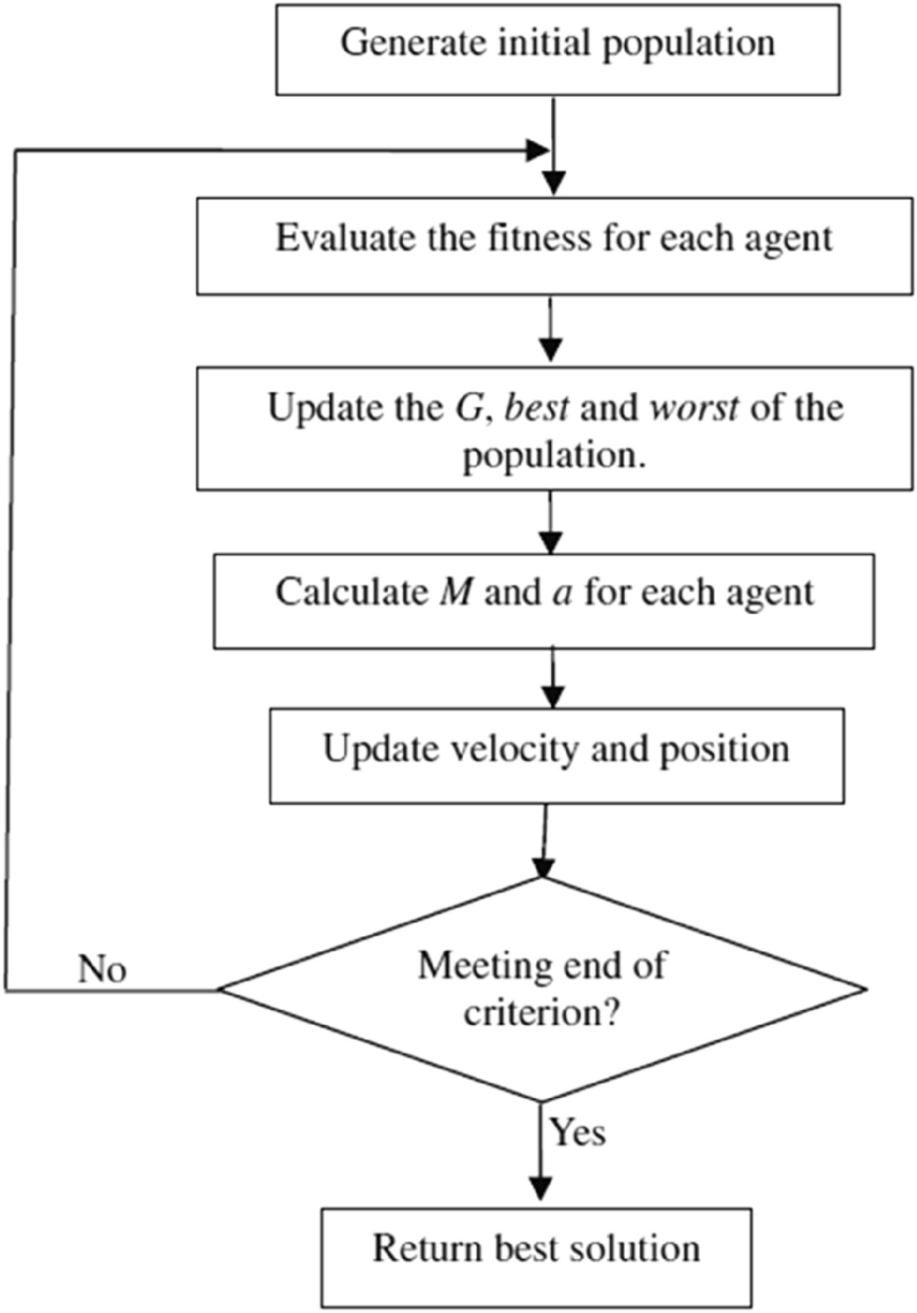
The Flow chart of the gravitational search algorithm procedures.

The Flow chart of the algorithm procedures is shown in Fig. 22. The heaviest object is presented with the problem solution according to the following steps:

- Search space identification.
- Initiate with random values.
- Evaluate the Fitness of all agents.
- Update the algorithm parameters: G (t), best (t), worst (t), and ${M}_{i}$(t) for i = 1, 2. . . N.
- The total force calculation in different directions took place.
- Calculating acceleration and velocity.
- Updating agents’ position.
- Repeat steps c to g until the stopping criteria are reached.
- End.

## Experimental results and discussion

This section discusses all experimental work done in this work. First, a study of the effect of eliminating the suck-back force on the accelerator performance is demonstrated. Then, an analysis of the impact of adding an iron yoke on the modified design is presented. In addition, the iron-yoked accelerator characteristics are illustrated and verified.

### Eliminating the suck-back force

As mentioned in the introduction, the suck-back force is a negative force acting on the projectile and should be eliminated. Suck-back force elimination depends on switching the accelerator coil off when the projectile’s centreline coincides with the accelerator coil’s centreline to prevent the coil from making an attraction force with the projectile toward inside the coil.

**Fig. 23:**
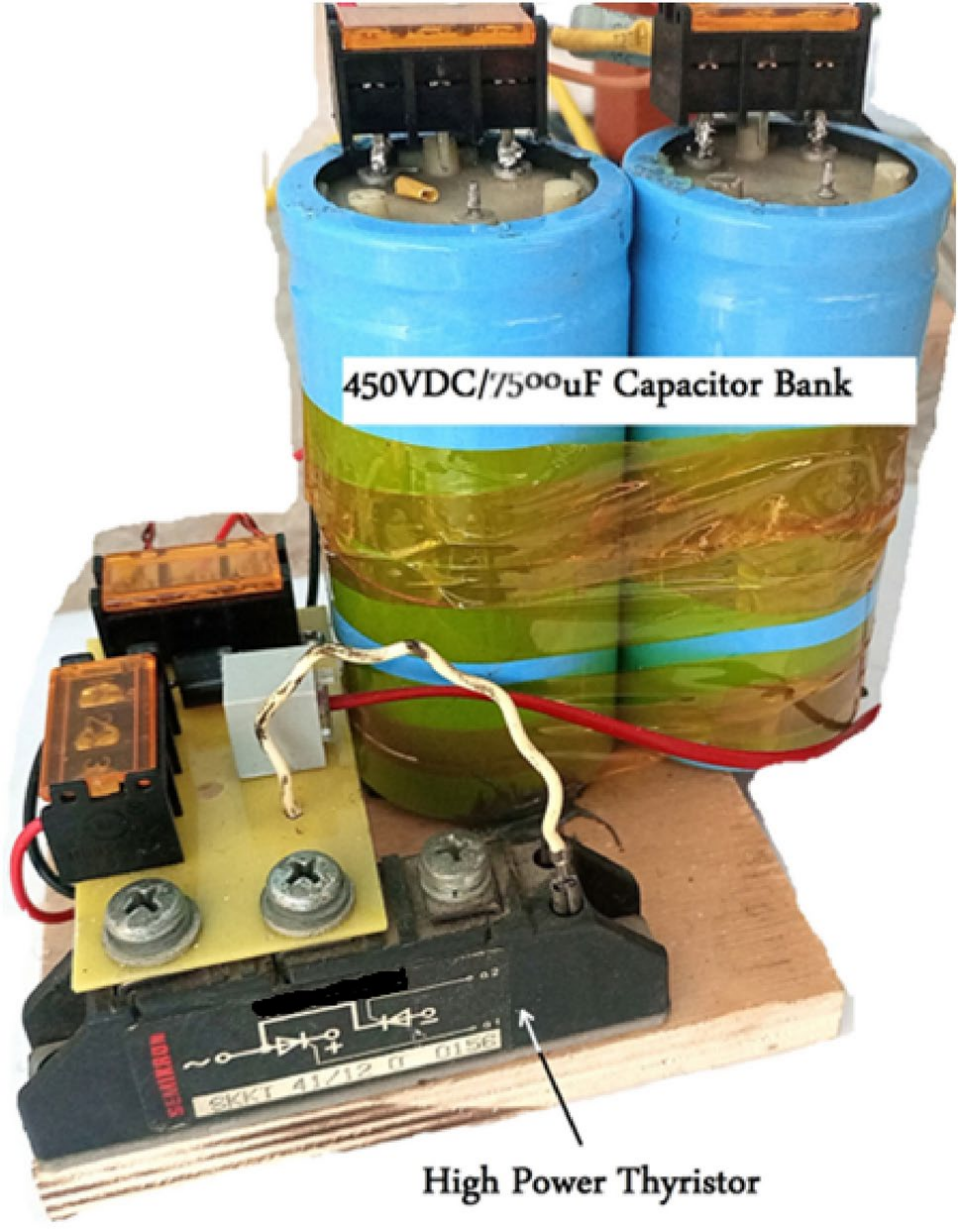
Thyristor-based switching module.

**Fig. 24:**
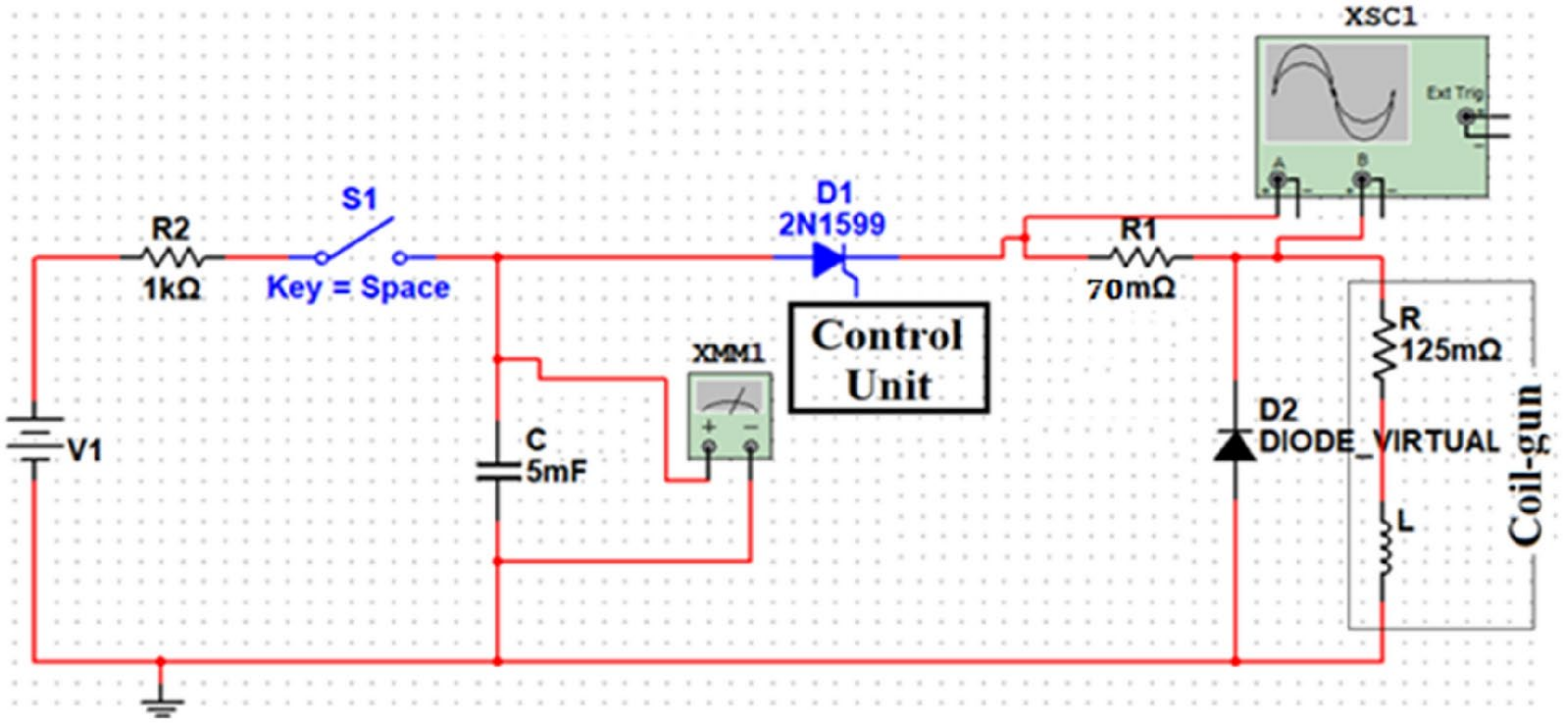
The system circuit has a thyristor as a switching device.

Two switching systems are examined to connect the coil with the capacitor bank to let the current form pass through the coil. The first one is the thyristor-based switching device (SEMIPACK SKKT 41/12), as shown in Figs. 23 and 24. This device can turn on the circuit and allow the current to flow easily, but to turn off the circuit and cut the current off, the force commutation method must be used, which is very costly, has a huge delay, and is complicated. Its gate can begin conduction but cannot be used to stop conduction. Meanwhile, the IGBT-based switching module, as shown in Figs. 6 and 15, has high controllability in the current waveform. Switching on and off is easier than the thyristor. IGBT is more suitable for high-frequency switching applications, while thyristor is better suited for high-power, high-voltage applications requiring AC power control.

**Fig. 25:**
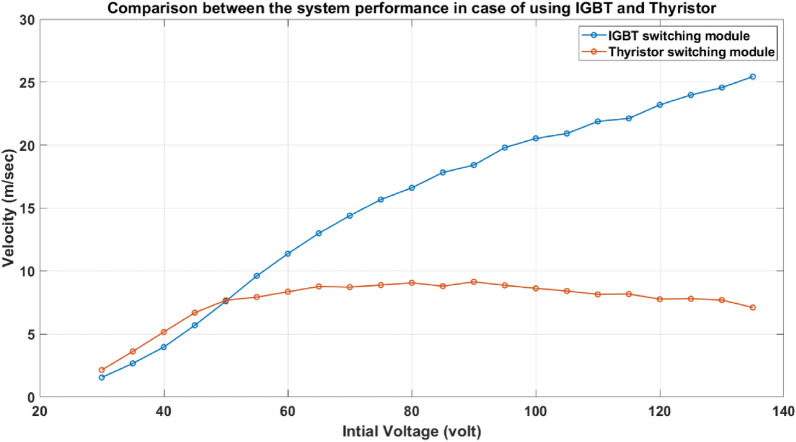
Comparison between the system performance in the case of using IGBT and Thyristor.

**Fig. 26:**
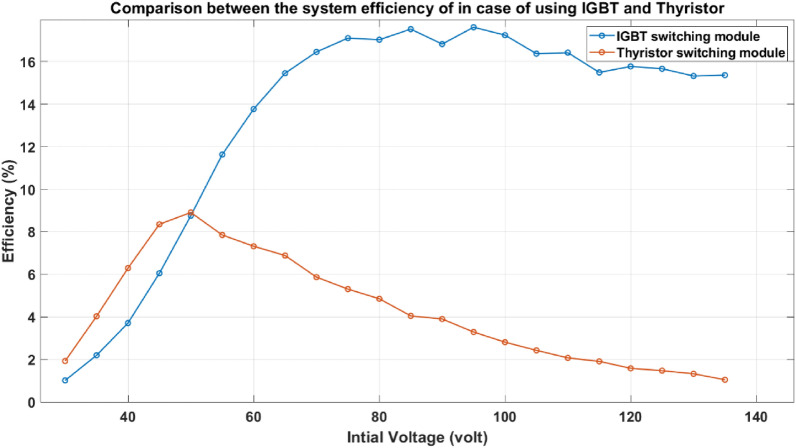
Comparison of the system efficiency in the case of IGBT and Thyristor.

To illustrate the difference between the two switching modules and their influence on the system performance and efficiency, the two modules are examined experimentally under the same conditions shown in Table 3 to control the experiments. Figure 25 shows the effect of using each module on the velocity trend for the accelerator system. As illustrated in the figure, the suck-back impact cannot be controlled in the thyristor-based switching system, which makes the velocity plateau at a certain limit even when the capacitor voltage is increased. On the other hand, the IGBT-based switching system can switch the coil off when the projectile’s centerline coincides with the accelerator coil centerline, which reduces the suck-back force effect and increases the velocity trend. In Fig. 26, the efficiency drops dramatically in the case of using the thyristor, while the efficiency rises in the case of using IGBT to more than 10 times than in the case of the thyristor. As a result, from this test, using IGBT is more recommended than using thyristor for that system.

**Table 3:** The experiment settings for the comparison between the thyristor and IGBT systems.

Projectile mass	Projectile and coil length	Projectile material	Number of turns	Yoke material
28.3 g	28 mm	soft iron	165	Soft iron

### Examination of the modified design

Many parameters control the number of magnetic flux lines. These techniques are clearly illustrated through Eq. (17), as it reveals that any rise in the current passes through the coil or the number of turns or reduction in the magnetic circuit reluctance results in a boosted pressure on the system to increase the number of the flux lines through the projectile^39^. Therefore, this work addresses the opposition of the magnetic reluctance to the magnetic flux produced by the accelerator coil. The problem is that the air around the accelerator coil represents a high magnetic reluctance due to low permeability, which leads to a drop in the magnetic flux, as demonstrated in Eq. (3). Accordingly, the authors add a high permeability iron yoke around the accelerator coil to create a new path for the flux inside the yoke, which drops the magnetic reluctance. The effect of the iron yoke is examined in the following sub-sections. The following experiments are controlled through the values in Table 4.

17 $$\varphi = \frac{NI}{{\Re_{{{\text{total}}}} }}$$

where: $\varphi$: magnetic flux lines within the magnetic material, ${\mathfrak{R}}_{\text{total}}$: The total magnetic reluctance of the accelerator magnetic circuit as a function of projectile position, $\text{N}$: Number of turns of accelerator coil, The current passes through the coil.

**Table 4:** The experiment setting for examination of The Modified Design.

Projectile mass	Projectile and coil length	Projectile material	Number of turns	Yoke material
28.3 g	28 mm	Soft iron	165	Soft iron

#### Inductance comparison

**Fig. 27:**
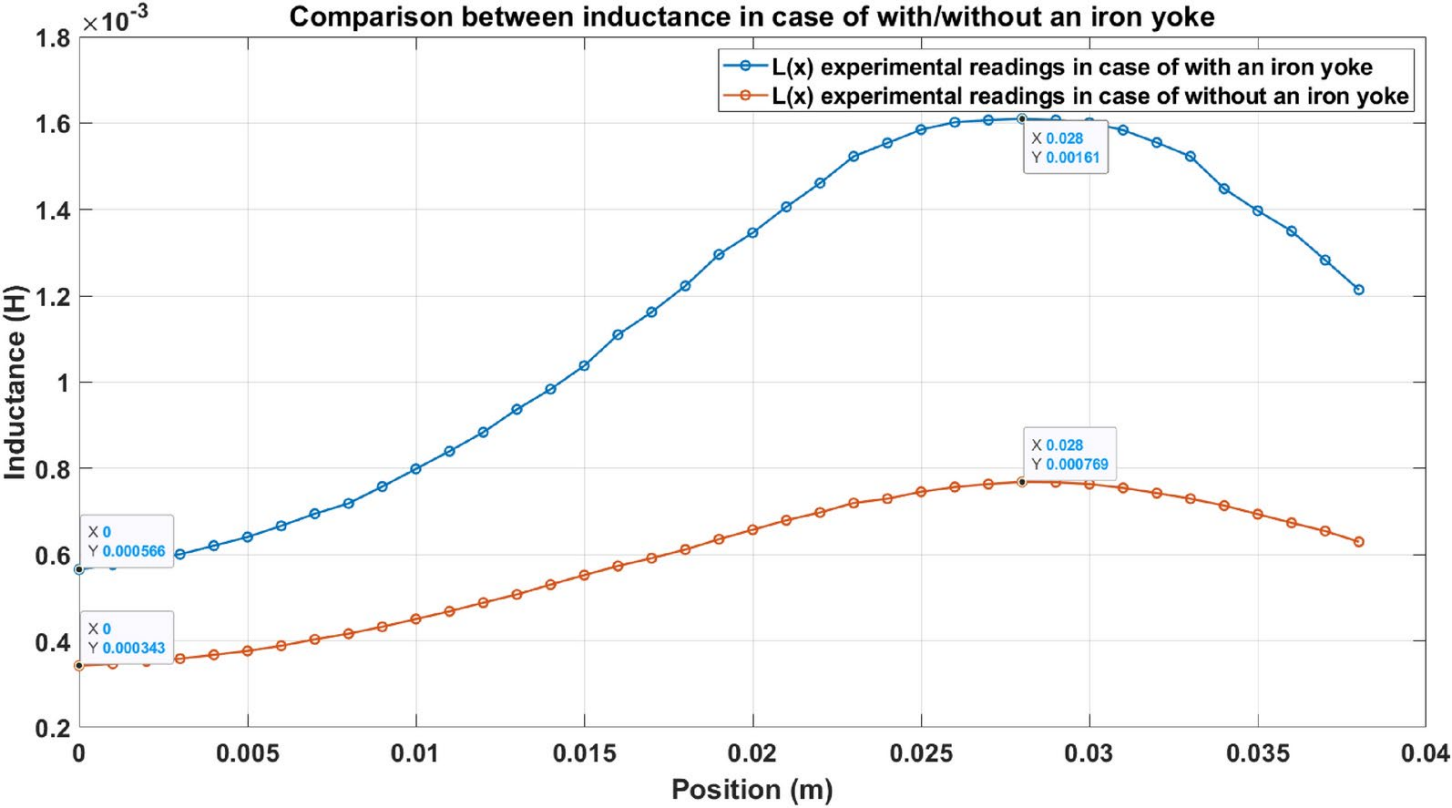
Comparison between inductance in the case of with/without an iron yoke.

**Fig. 28:**
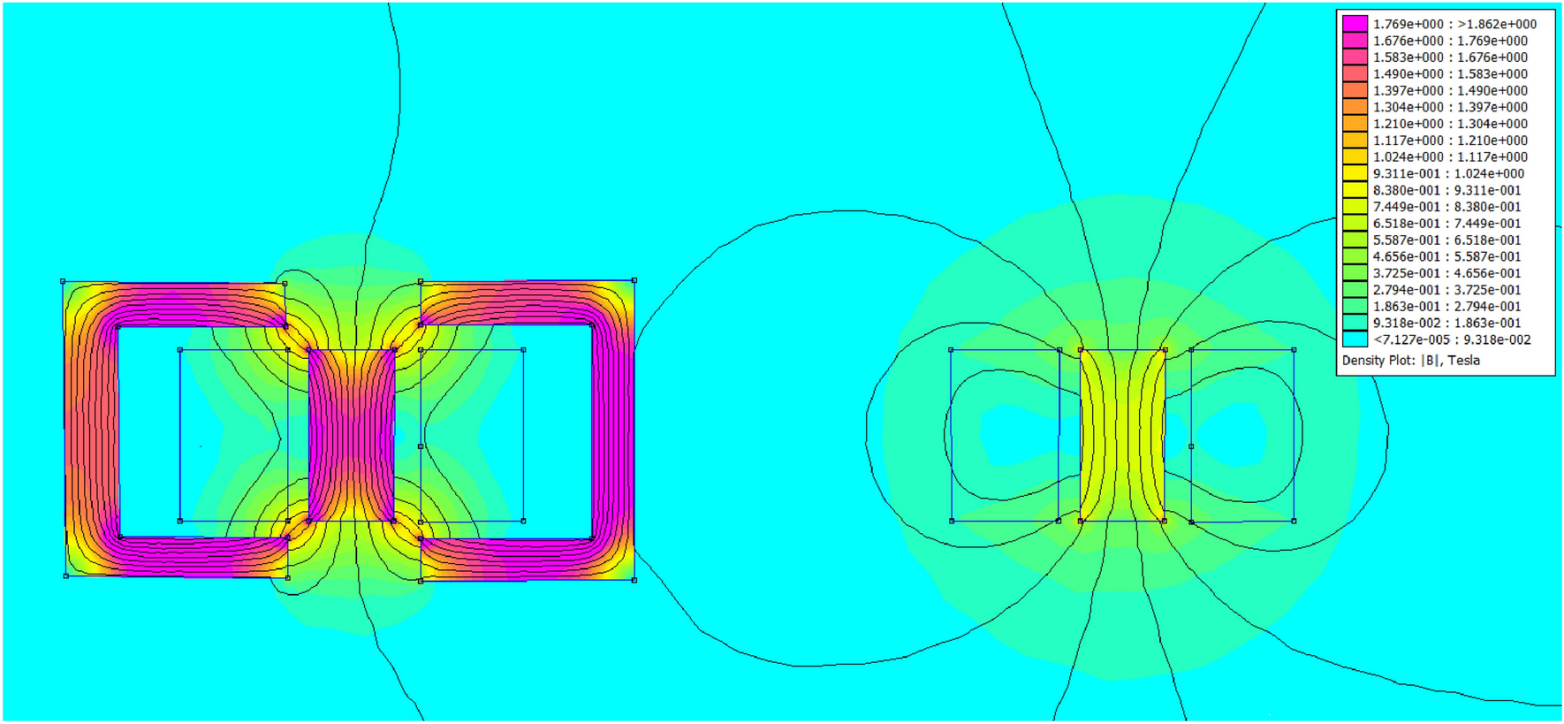
The magnetic flux lines contour in a case with /without the iron yoke.

This part investigates the influence of adding a high-permeability iron yoke on the coil inductance. According to Eq. (4), the coil inductance is inversely proportional to the resultant magnetic reluctance, and to clarify and study this effect, a comparison between two identical accelerators is performed, but one has an iron yoke, and the other doesn’t, to control the experiment as shown in Fig. 3. The used iron yoke consists of 92 laminations of soft iron to reduce the eddy current produced. The result displayed in Fig. 27 shows that the inductance of the iron-yoked accelerator doubles at its peak compared to the inductance in the absence of the iron yoke. Moreover, the iron-yoked accelerator’s initial value is about 65% higher than the initial value of the inductance in the absence of an iron yoke. To understand the effect deeply, the magnetic flux lines are plotted by using FEMM4.2 software when the coil current equals 200 A, as shown in Fig. 28. That figure shows the magnetic flux density in the case of with/without the iron yoke and illustrates that the iron yoke allows more flux lines to pass through the projectile. That leads to a high slope $(\frac{\Delta L}{\Delta x})$ that directly affects the developed force on the projectile, as proved in^22,40^ and shown in Eq. (15). However, adding the iron yoke improves the performance; the saturation of this iron yoke will limit the allowed coil’s current and the number of flux lines. That’s why the iron yoke used is made of soft iron sheets with a magnetic density limit of B = 1.8 Tesla, which is specified in the manufacturer’s B-H curve, as displayed in Fig. 18.

#### Inductance derivative comparison

**Fig. 29:**
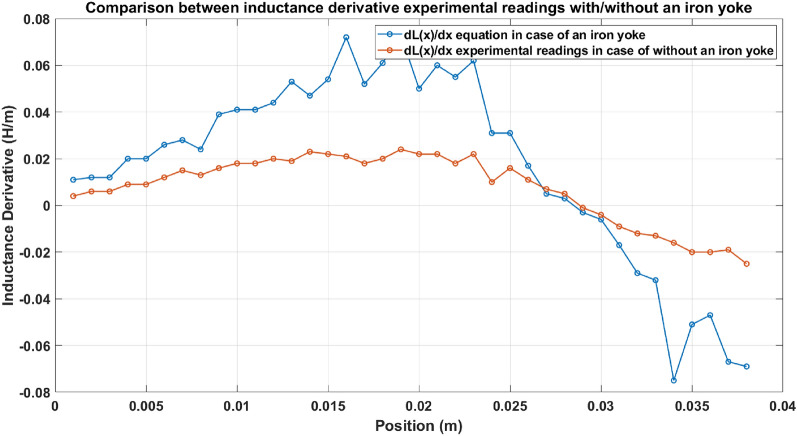
Comparison between inductance derivative experimental readings in the case of with/without an iron yoke.

The developed force is strongly related to the change in the coil inductance concerning the projectile position $(\frac{\Delta L}{\Delta x})$ as shown in Eq. (15). Therefore $\left(\frac{\Delta L}{\Delta x}\right)$ of an iron-yoked accelerator is compared with $\left(\frac{\Delta L}{\Delta x}\right)$ of an ordinary accelerator (without an iron yoke), as displayed in Fig. 29 to ensure the modification. Figure 29 shows the superiority of the iron-yoked accelerator over the regular accelerator in producing higher $\left(\frac{\Delta L}{\Delta x}\right)$ and, consequently, higher accelerating force.

#### Accelerator performance and efficiency

**Fig. 30:**
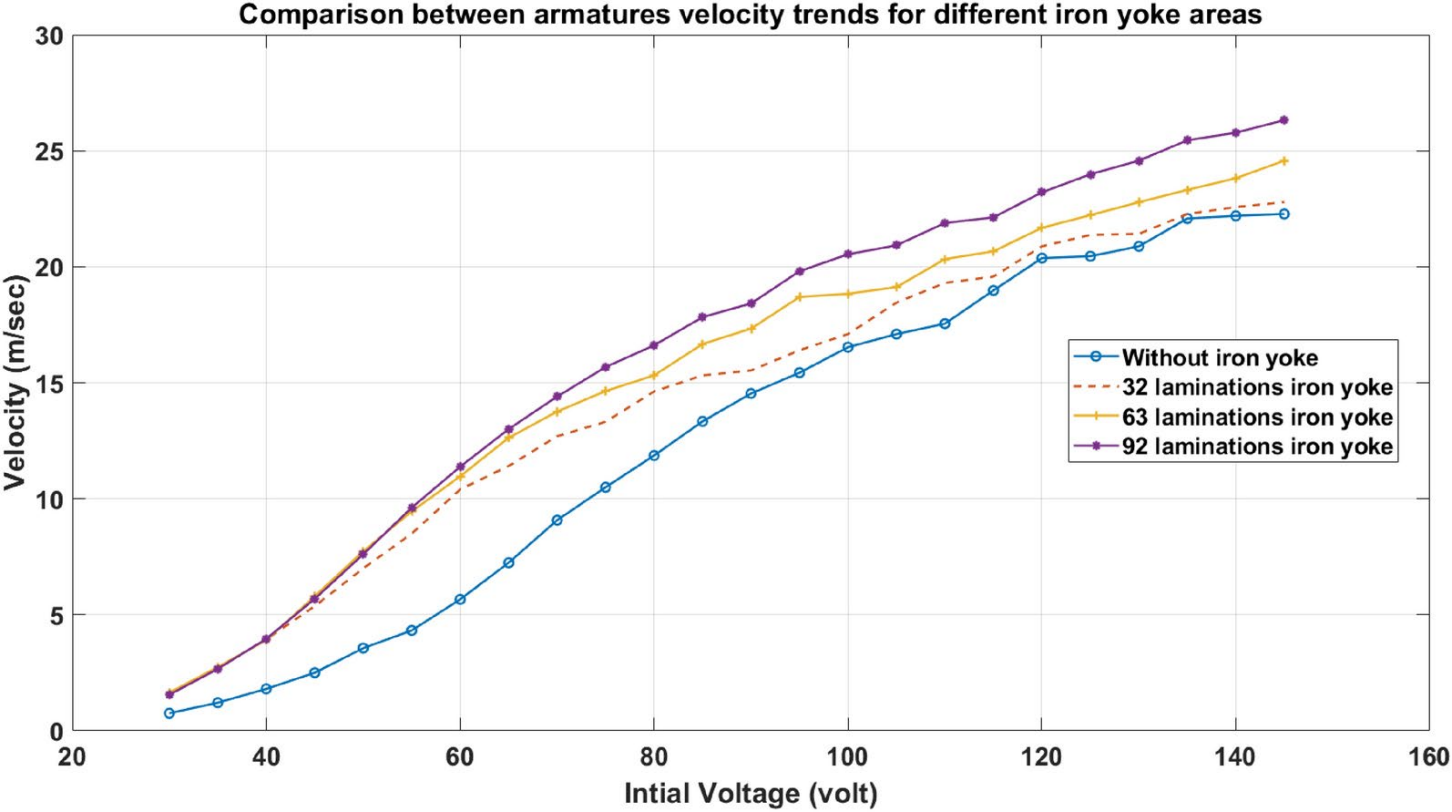
Velocity readings in the case with/without an iron yoke.

**Fig. 31:**
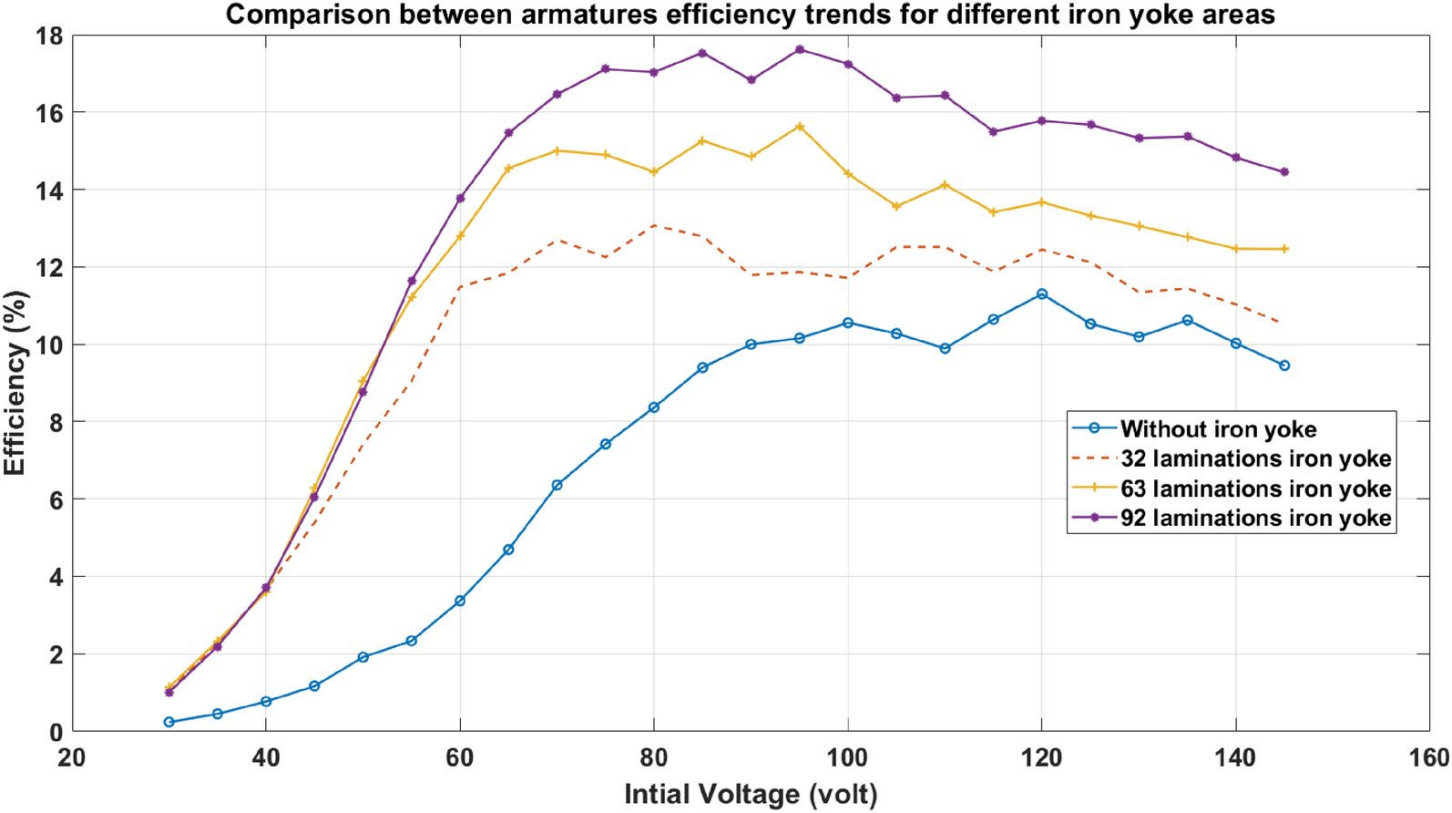
Accelerator efficiency in case of with/without an iron yoke.

In this part, three different widths of the iron yoke are examined to illustrate the effect of adding an iron yoke. A graph showing the variation of voltage and projectile velocity is plotted to demonstrate the performance of the accelerator in four cases (the three widths iron-yoked accelerator and the ordinary accelerator) and shown in Fig. 30. Figure 30 shows that as the width of the iron yoke increases, the accelerator’s performance is enhanced. When the area of the iron yoke increases, more magnetic flux lines pass through it. In addition, the velocity trend of the 92-lamination iron-yoked accelerator is higher than that of the ordinary accelerator of the same dimensions and design by about 30% at the best efficiency points. This proves that adding an iron yoke reinforces the accelerator’s performance. Furthermore, the efficiency of each accelerator is determined as illustrated in Fig. 31 and calculated by Eq. (18). To clarify the improvement done due to boosting the number of flux lines resulting from the addition of iron yoke, the efficiency of the modified accelerator is about 60% higher than the efficiency of the regular accelerator, which is an encouraging percentage that makes this accelerator promising.

18 $${\text{Efficiency}} = { }\frac{{\frac{1}{2} m v^{2} }}{{\frac{1}{2} C \left( {V_{intial}^{2} - V_{final}^{2} } \right)}}$$

where: $v$: Projectile velocity, $m$: Projectile mass, $C$: Capacitor bank capacity value, ${V}_{intial}$: The initial voltage of the capacitor bank, ${V}_{final}$: The final voltage remains across the capacitor bank after propelling.

### Iron-yoked accelerator characteristics

To verify that adding an iron yoke to the accelerator will not negatively influence performance. Two propulsion system characteristics should be examined. The values in Table 5 control the following two experiments.

**Table 5:** The experiment setting for Iron-yoked accelerator characteristics examination.

Projectile mass	Projectile and coil length	Projectile material	Number of turns	Yoke material
28.3 g	28 mm	Soft iron	165	Soft iron

#### Repeatability test

**Fig. 32:**
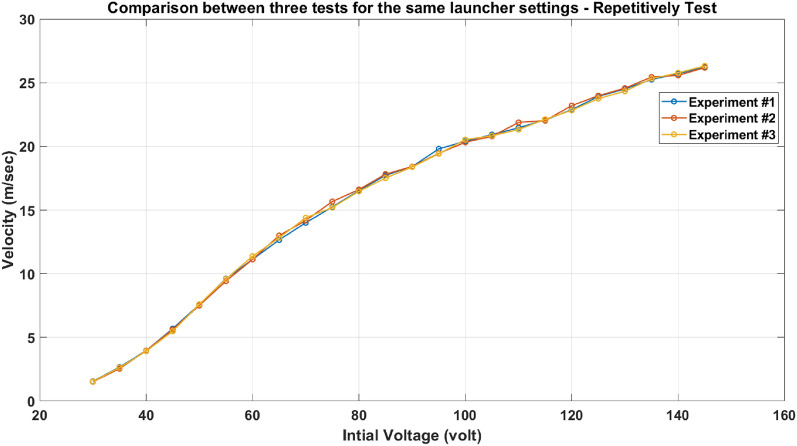
Three velocity trends of the iron-yoked accelerator for three successive experiments.

This test checks the system’s reliability by giving the same velocity multiple times for the same voltage after adding an iron yoke. All system settings are constant for the three experiments to control the test. As shown in Fig. 32, three successive experiments’ velocity trends are plotted, and the three curves are approximately identical. These experiments prove that the iron yoke does not affect the velocity for the same capacitor voltage each time the accelerator is used at the same conditions.

#### Hysteresis test

**Fig. 33:**
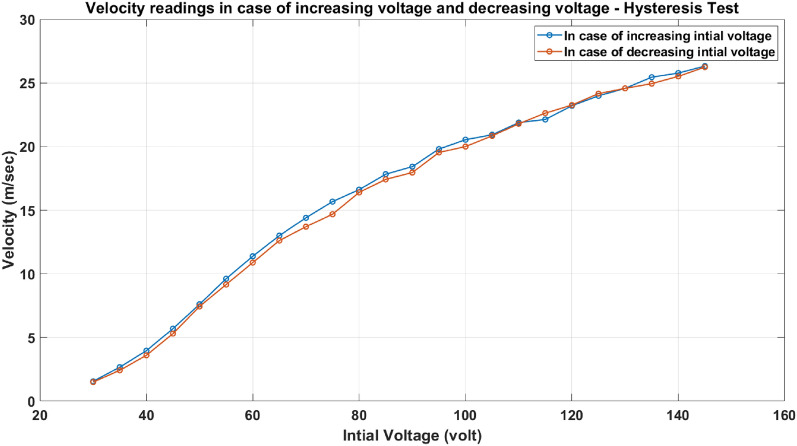
The velocity trend in case of increasing the applied voltage (Forward increase) and in case of decreasing the applied voltage (Backward increase).

Hysteresis occurs in a system that involves a magnetic field. Hysteresis is a common property of ferromagnetic substances. Two experiments are carried out to ensure the iron yoke substance is not magnetized. The velocity trend is measured experimentally, as displayed in Fig. 33, in the case of increasing the applied voltage (Forward increase) and decreasing the applied voltage (Backward increase). Figure 33 This shows that adding a laminated iron yoke will not affect the accelerator performance when the voltage increases or decreases.

#### Comparison between cheetah optimizer and gravitational search algorithm

This section proposes comparing the Cheetah Optimizer and gravitational search algorithm to optimize the accelerator system. To control the comparison, both techniques are settled on the same parameter values, as shown in Table 6. For the same lower and upper limits, Table 7 shows the results of each method. The GSA-optimized accelerator is more efficient than the CO-optimized one, and it selects convenient parameters that give it 2.7% higher efficiency than the CO-optimized one. Therefore, further studies will be completed with GSA.

**Table 6:** The CO and GSA parameters.

CO parameters	GSA parameters
Population Size = 30	Number of agents: 30
Max number of iterations = 10	Max number of iterations = 10
Processor: Core i5 9th generation, 3.98GHz	Processor: Core i5 9th generation, 3.98GHz

**Table 7:** Lower and upper limits for CO and GSA algorithms and the optimization results.

	Volt	Capacitor	Number of turns	Efficiency
Upper limit	115	8000 uF	175	
Lower limit	95	4000 uF	150	
CO results	101.4309	5500 uF	165.6836	21.23%
GSA results	108.5624	4300 uF	164.7888	21.8%

#### GSA Optimization results and examination

**Fig. 34:**
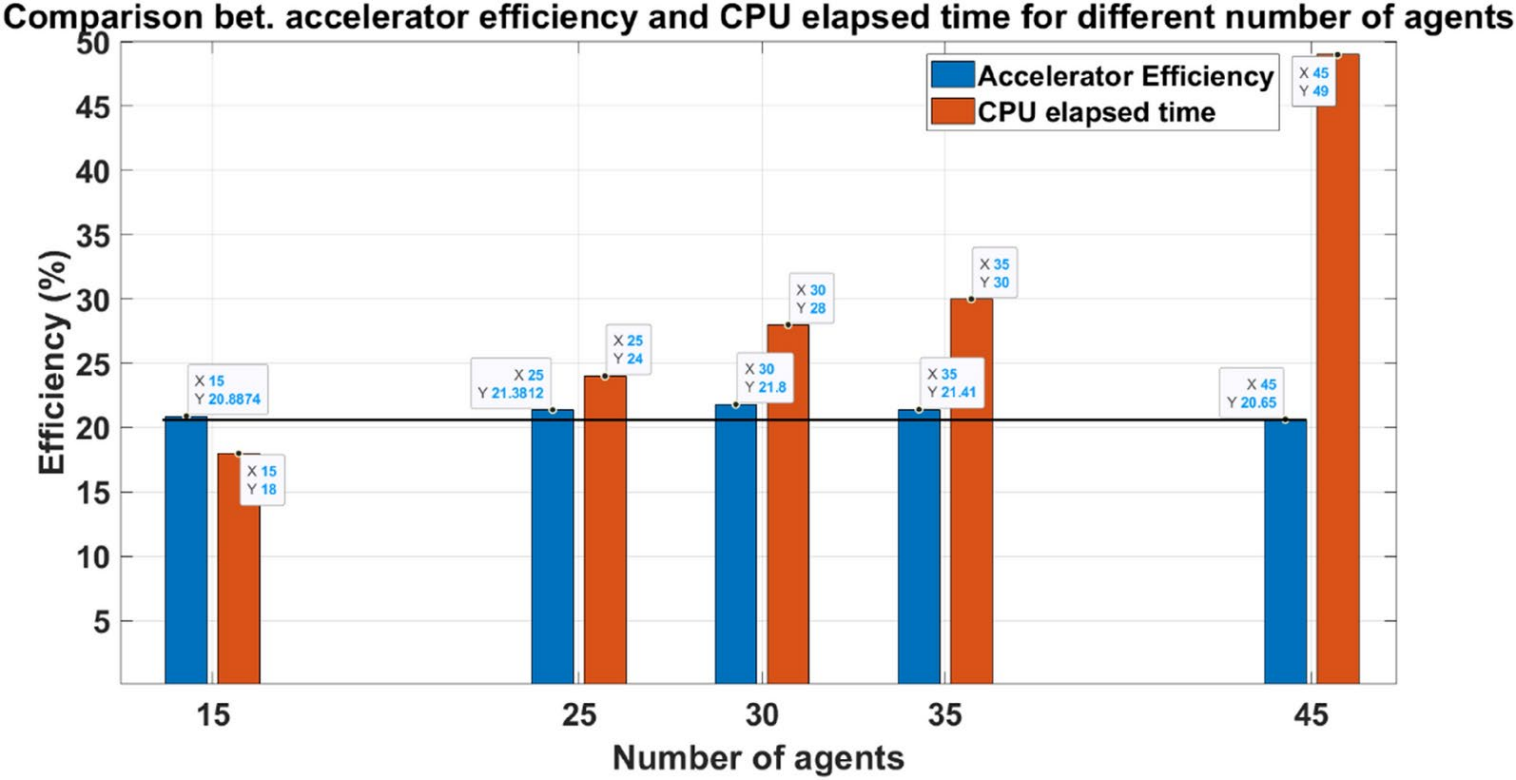
Accelerator efficiency and CPU elapsed time for different numbers of agents.

The MATLAB program applies the GSA optimization technique to the accelerator system model. In this work, the max number of iterations is fixed (max number of iterations = 10) with a different number of agents to examine its effect. The results shown in Table 8 and Fig. 34 are deduced when the GSA code runs on a core i5 9th generation, 3.98GHz processor. GSA gives good results over a wide range of agents, but the proper number of agents is 30, which offers a local maximum among the examined values. Therefore, in this work, the GSA-optimized parameters are selected at a number of agents = 30, as shown in Table 9. GSA*’*s tasks in this work are obtaining the optimal capacitor value, the optimal number of turns, and the maximum efficiency point regarding the saturation of the iron yoke.

**Table 8:** The efficiency of the accelerator for the gsa-optimized parameters for different numbers of agents.

Number of agents	Efficiency (%)	Elapsed time (mins)
15	20.8874	18
25	21.3812	24
30	21.8	28
35	21.41	30
45	20.65	49

**Table 9:** GSA Results and the actual values applied according to the standard.

	Volt	Capacitor (uF)	Number of turns
GSA results	108.5624	4300	164.7888
Actual values	110	5000	165
Upper limit	115	8000	175
Lower limit	95	4000	150

**Fig. 35:**
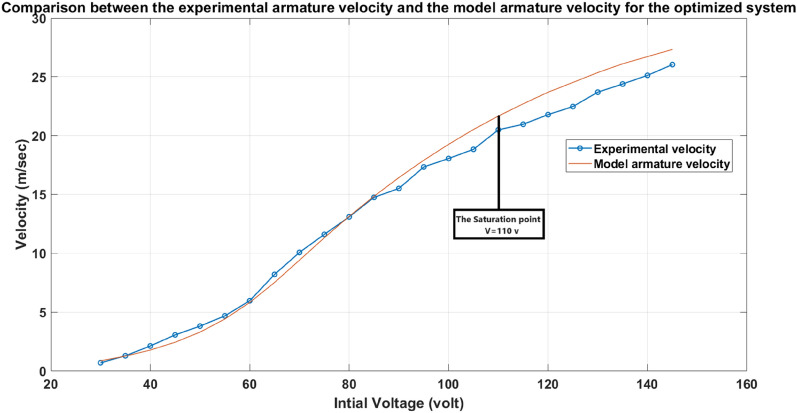
The experimental armature velocity and the model armature velocity for the optimized system.

All the experiments and studies are performed using the accelerator system with trial-and-error selected parameters. Therefore, the optimized system performance is compared with that old system to examine the power of GSA in choosing the optimal parameters. The optimized system parameters are mentioned in Table 9. The GSA obtained values in Table 9 (First row) are difficult to obtain practically. Therefore, the nearest standard values are used (Second row). Another validation for the SIMULINK model, the optimized system experimental results are compared with the SIMULINK model results as shown in Fig. 35.

**Fig. 36:**
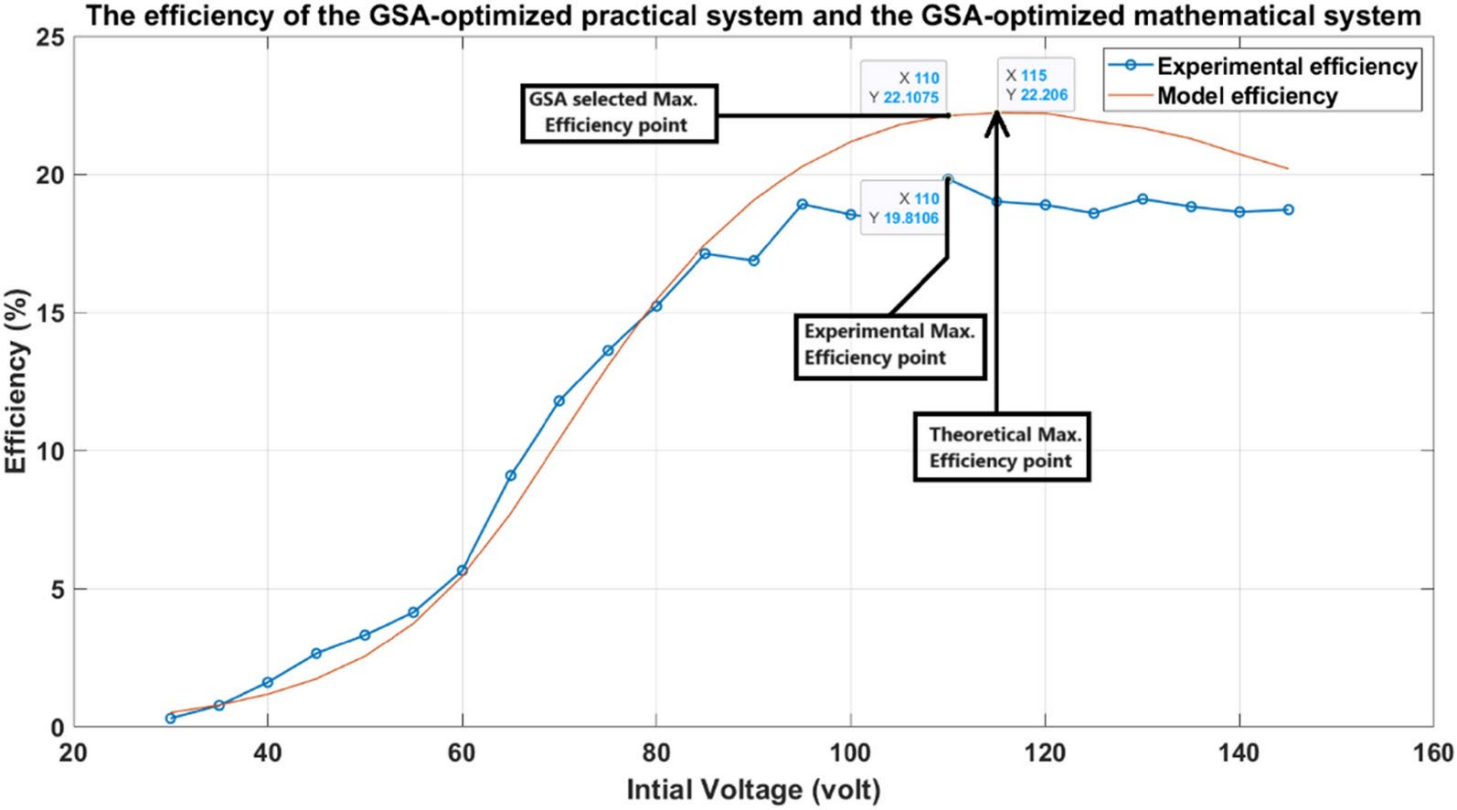
The efficiency of the GSA-optimized practical system and the GSA-optimized mathematical system.

Figure 36 shows that the theoretical maximum efficiency point is at 115v. At the same time, the GSA technique determines the maximum efficiency point at 108.5 v when the saturation of the iron yoke is considered. This is approximately the same as the experimental maximum efficiency point, which is at 110v.

**Fig. 37:**
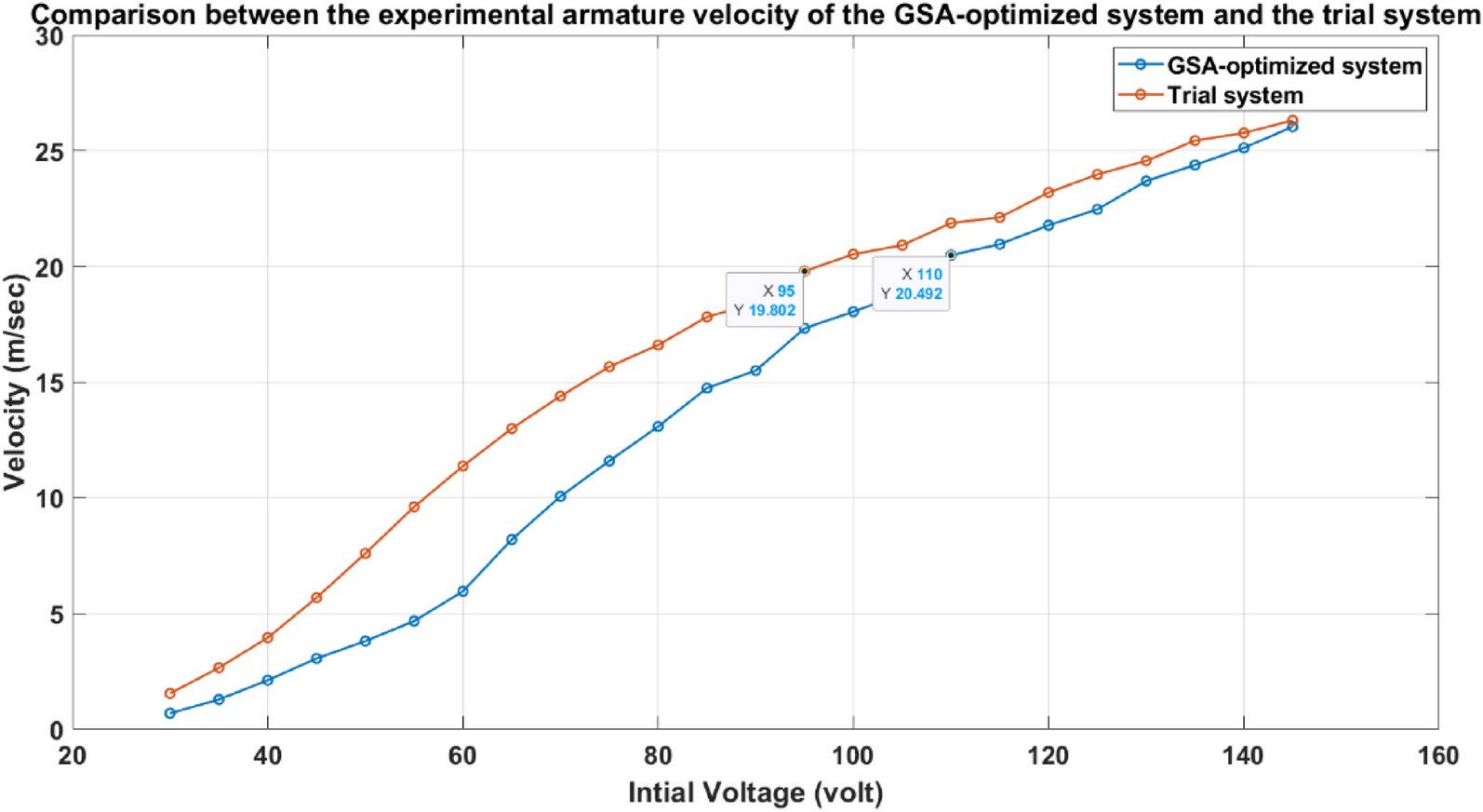
The experimental armature velocity of the GSA-optimized system and the trial system.

**Fig. 38:**
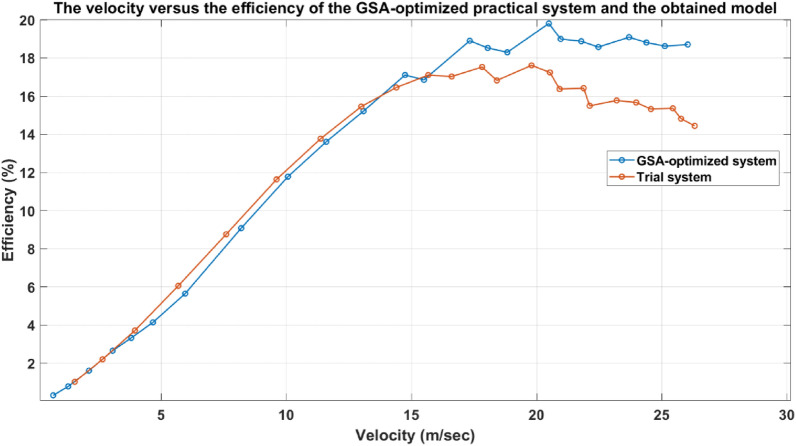
The velocity versus the efficiency of the GSA-optimized practical system and the obtained model.

In Fig. 37, the velocity trend in the case of the optimized accelerator has a higher slope than the velocity trend of the system before applying the optimized parameters (un-optimized system), which proves its better performance. As shown in Fig. 38, the efficiency of the accelerator of the optimized system is higher than the efficiency of the old system for the same velocity. In other words, to get a certain projectile velocity, the optimized system offers higher efficiency than the old system, especially at higher velocities (over 16 m/sec.). The settings of all experiments in this part are mentioned in Table 10. The maximum efficiency of the un-optimized system is about 17.5% at a velocity of 19.8 m/sec, while the optimized system achieved about 20% efficiency at a velocity of 20.492 m/sec, representing a 15% increase in the GSA-optimized system’s efficiency. The max velocity of both systems is about 26 m/sec, but the efficiencies are different; optimized system efficiency equals 18.5%, while the un-optimized system efficiency equals 14.5%.

**Table 10:** The GSA-Optimized System Settings.

Projectile mass	Projectile and coil length	Projectile material	Number of turns	Yoke material	Capacitor
28.3 g	28 mm	Soft Iron	165	Soft Iron	5000 uF

**Fig. 39:**
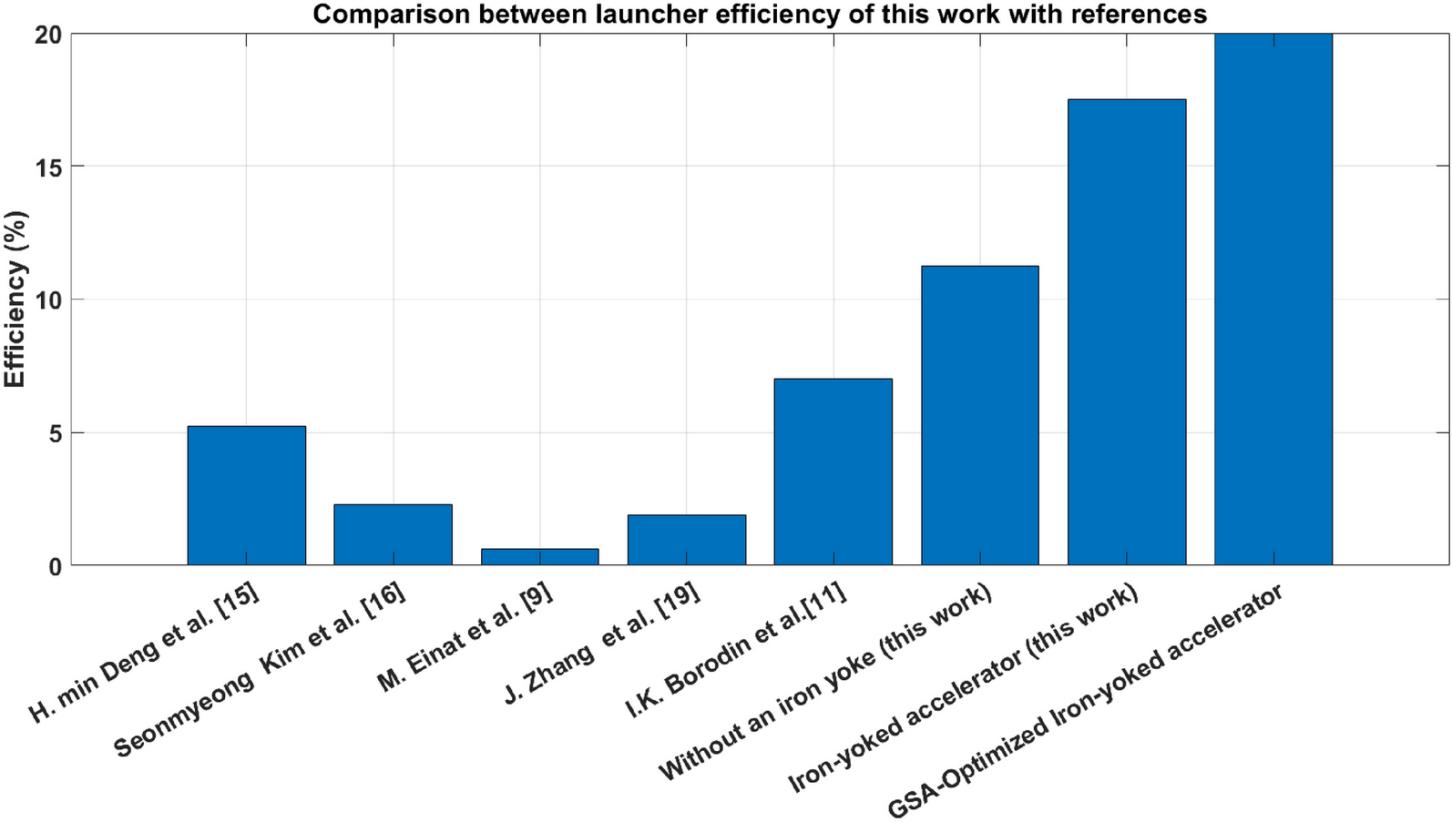
Comparison between launcher efficiency of this work with references.

The effect of adding an iron yoke to the accelerator coil and the modified system efficiency are illustrated and compared with some literature works, as displayed in Table 11 and Fig. 39. This work presents two systems, one with an iron yoke and the second without an iron yoke. Notably, the suck-back force is eliminated in both systems by using an IGBT switching module with continuous feedback to turn off the system when the projectile’s centreline coincides with the coil’s centreline. Table 11 shows that many works of literature have lower efficiency than the proposed accelerator design results, as plotted in Fig. 39. This proves the essentiality of eliminating the suck-back force and the role of reducing the magnetic reluctance by an iron yoke. Finally, using GSA to optimize the system is essential in choosing the optimized system parameters.

**Table 11:** Comparison between the proposed work results and the literature works.

Refs. no	Used voltage (Volt)	Max. Velocity ($\frac{{\varvec{m}}}{ {\varvec{s}}{\varvec{e}}{\varvec{c}}}$)	Mass (g.)	Eff. (%)	Year
H. min Deng et al.^18^	500	8.24	338	5.25	2020
Seonmyeong Kim et al.^19^	400	31	11.35	2.27	2022
M. Einat et al.^13^	900	33.8	2.5	0.597	2023
J. Zhang et al.^22^	156	9.8	4.36	1.9	2023
I.K. Borodin et al.^11^	–	80	19,600	7	2024
**Accelerator without an iron yoke**	**145**	**21**	**28.3**	**11.25**	**2024**
**Iron-yoked accelerator**	**145**	**26**	**28.3**	**17.5**	**2024**
**GSA-Optimized Iron-yoked accelerator**	**145**	**26**	**28.3**	**20**	**2024**

## Contributions and the future work

### Contributions

The paper contributes to the research society through the following:

1. Innovative Accelerator Design Modification: A novel and reliable accelerator design is developed, specifically targeting and addressing challenges outlined in the existing literature.
2. Deduction of Mathematical Model: A thorough mathematical model is derived to clarify the fundamental principles governing the accelerator system.
3. System Implementation and Fabrication: The proposed system is designed and built from the ground up, ensuring a comprehensive approach to its development.
4. Simulation and Verification via SIMULINK Model: The developed SIMULINK model is simulated, and its performance is experimentally validated.
5. Experimental examination of GSA-Optimized System: The system, optimized using a gravitational search algorithm (GSA), undergoes thorough experimental assessment, and its performance is compared against the prior design and existing literature.

### Suggestions for future work

The following directions are suggested:

- Redesign a more significant accelerator to propel a larger projectile.
- Investigate modified magnetic materials with higher saturation.
- Different optimization techniques like particle swarm should be used.
- Use multi-objective optimization.
- Ultra-fast sensors and processors can be utilized.
- Build a multi-iron-yoked-coils accelerator and investigate its performance.
- Optimize the number of cascaded coils that give the highest efficiency.
- Use this accelerator as a propulsion system for meg-lev trains.
- Engage the accelerator system with renewable sources of energy.

## Conclusion

This paper presents a modified design for a coil electromagnetic accelerator to enhance efficiency and performance in propelling ferromagnetic projectiles. The main innovation is encircling the accelerator coil with a soft iron yoke, which increases magnetic flux and lowers magnetic circuit reluctance. Performance is further enhanced by removing the suck-back force through a fast Insulated Gate Bipolar Transistor (IGBT) module, continuous feedback mechanisms for the projectile’s position, and a dependable control system. It has been demonstrated that the modified accelerator system—which consists of an iron-yoked accelerator coil, a bank of capacitors, an IGBT module with continuous feedback, and a 133 MHz Dual-core Arm Cortex processor-based control system—performs better than conventional accelerators. Despite a slight increase in mass, the modified design achieves a significant efficiency improvement of 60%, reaching 17.5% compared to 11.25% for traditional designs without a suck-back force. Moreover, this design makes the inductance value of the iron-yoked accelerator twice the inductance in case of the absence of the iron-yoke at its peak. Additionally, the initial inductance of the iron-yoked accelerator is approximately 65% higher than that of the coil without the iron yoke. The efficiency of this modified design is almost three times higher than the efficiency of 5.95%, which was reported in the latest studies, indicating its superiority. In addition, the properties of iron-yoked accelerators were examined, showing how the additional iron yoke helps to direct the magnetic flux and enables a more refined mathematical model. This model’s MATLAB simulation aligned excellently with the experimental data, with a maximum variance of just 4.48%. This verification of the model’s accuracy provides trust in the effectiveness of the design.

Additionally, the number of turns, capacitor value, and capacitor voltage were optimized—three crucial accelerator parameters—by using the gravitational search algorithm (GSA). The GSA-optimized system demonstrated a 15% increase in efficiency compared to the unoptimized system in the experimental evaluation, demonstrating the potential of artificial intelligence techniques in improving the performance of electromagnetic accelerators. The study’s overall conclusions demonstrate how well the modified iron-yoked design enhances electromagnetic acceleration. Further studies could explore additional system parameter optimization and the design’s scalability for varying projectile sizes and materials.

## Data Availability

*Most data generated or analyzed during this study are included in this published article. *However, some datasets used and/or analyzed during the current study are confidential and available from the corresponding author upon reasonable request.
